# Transferring cognitive talent across domains to reduce the disposition effect in investment

**DOI:** 10.1038/s41598-021-02596-2

**Published:** 2021-11-29

**Authors:** Kristian Rotaru, Petko S. Kalev, Nitin Yadav, Peter Bossaerts

**Affiliations:** 1 Monash Business School, Monash University, Caulfield East, VIC 3145 Australia; 2 BrainPark, The Turner Institute for Brain and Mental Health, School of Psychological Sciences and Monash Biomedical Imaging Facility, Monash University, Clayton, VIC 3800 Australia; 3 Department of Economics, Finance and Marketing, La Trobe Business School, La Trobe University, Bundoora, VIC 3086 Australia; 4 Brain, Mind and Markets Lab, University of Melbourne, Parkville, VIC 3050 Australia; 5 Faculty of Economics, University of Cambridge, Cambridge, CB3 9DD UK

**Keywords:** Human behaviour, Social behaviour, Decision, Empathy

## Abstract

We consider Theory of Mind (ToM), the ability to correctly predict the intentions of others. To an important degree, good ToM function requires abstraction from one’s own particular circumstances. Here, we posit that such abstraction can be transferred successfully to other, non-social contexts. We consider the disposition effect, which is a pervasive cognitive bias whereby investors, including professionals, improperly take their personal trading history into account when deciding on investments. We design an intervention policy whereby we attempt to transfer good ToM function, subconsciously, to personal investment decisions. In a within-subject repeated-intervention laboratory experiment, we record how the disposition effect is reduced by a very significant 85%, but only for those with high scores on the social-cognitive dimension of ToM function. No such transfer is observed in subjects who score well only on the social-perceptual dimension of ToM function. Our findings open up a promising way to exploit cognitive talent in one domain in order to alleviate cognitive deficiencies elsewhere.

## Introduction

**Figure 1:**
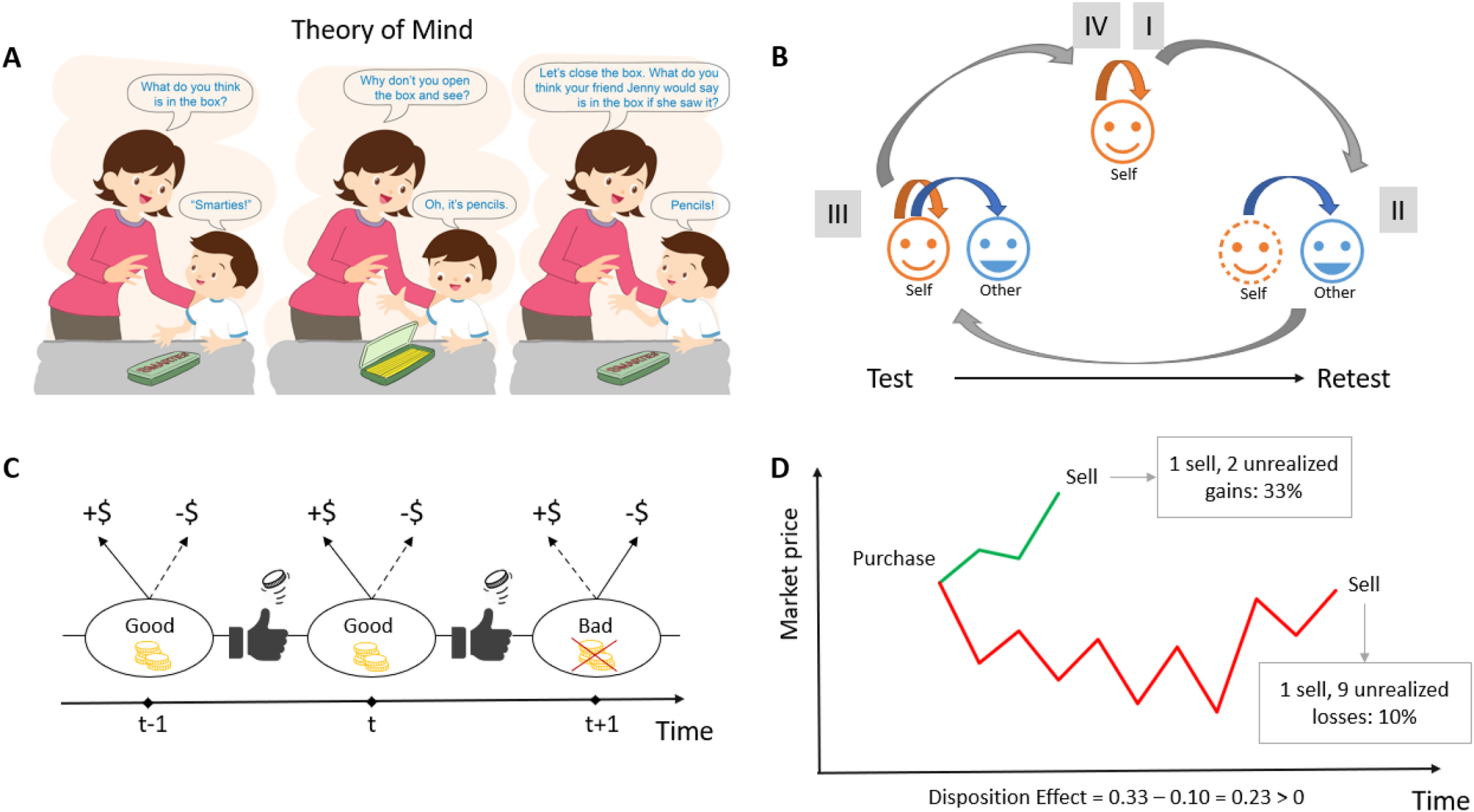
(**A**) ToM is the ability to abstract from one’s own experience to correctly guess the mindset of another. Here, the child incorrectly abstracts from its knowledge that the box contains pencils rather than smarties when predicting Jenny’s response^5^. Hence, the child has incorrect ToM. (**B**) Structure of one sitting in the experiment: (I) and (IV) Participant plays an investment game for self; (II) Participant plays the game for someone other, who never remains invested more than one round; (III) Participant makes choices for self and for other. (**C**) Stock price changes are more likely to be positive in the good regime; conversely, stock price changes are more likely to be negative in the bad regime; regime switches are random across rounds, determined by a biased coin flip. Under the Bayes-optimal strategy, gains rarely are realized, while losses are realized regularly. (**D**) Computation of the DE (Disposition Effect) metric: under the green price history between Purchase and Sale, gains were not realized for 2 rounds; under the red price history between Purchase and Sale, losses were not realized 9 rounds. The metric equals the difference between the percentage of periods with realized gains relative to all periods with gains (paper gains or realized gains) and the percentage of periods with realized losses relative to all periods with losses. In the example here, the player hangs on to losses much longer, and hence, DE is positive.

A subdomain of a broader concept of social cognition^1,2^, Theory of Mind (ToM) refers to the ability to impute mental states to others that may be different from one’s own^3–5^ (Fig. 1A). Two components of ToM are commonly distinguished: *social-perceptual ToM* refers to the ability to perceive, or “decode”, mental states of others based on directly observable knowledge; whereas *social-cognitive ToM* is associated with the ability to infer the underlying mental states of others by observing their behaviors^4,6^. To be good at ToM, abstraction is required. One’s own personal circumstances are to be abstracted from in order to better gauge the intentions of others^7,8^. This is of utmost importance in many situations, such as strategic interaction^9^. But abstraction is needed not only in social situations. Here, we consider investments in the context of finance.

One of the most pervasive cognitive biases affecting investors, even professionals, is the disposition effect (DE)^10,11^. This bias emerges when an investor hangs on to losses too long while selling assets too quickly after gains. Losses and gains are defined with respect to the price at which the investor acquired the position: a loss (resp. gain) is incurred when the current market price is below (resp. above) the acquisition price. Since market prices evolve irrespective of actions from a single individual, rational investment decisions rely solely on future prospects, not on a person’s historical experience. For instance, personal losses incurred in the recent past merely amount to “sunk costs,” and hence are irrelevant. To avoid DE, it is of primordial importance to be able to abstract away from one’s own history. Therefore, avoidance of DE and ToM have one aspect in common: the ability to abstract.

It deserves emphasis that our linking ToM with abstract thinking is not without controversy. Conscious experience provides an alternative way to acquire the capacity to predict the intentions of others (e.g. Johnson, 1988^12^). Conscious experience is concrete, not abstract. It does not require a “theory” or a “model” with which intentions could be deduced given contextual signals. Conscious experience relies on awareness that one’s experience in a particular context applies to others as well. Mathematically, one could model it as an exercise in projection. Because there are various plausible ways to explain ToM function, our training scheme effectively relies on a joint hypothesis, namely, that transfer of abstraction to non-social domain is possible, and that ToM function builds on abstraction.

Many researchers do link ToM and abstract thinking explicitly. For instance, Tenenbaum and collaborators (2011, p. 1284)^13^ mentioned ToM as an example of “Bayesian thinking”, explaining that, at its core, Bayes’ law is a tool to allow abstract knowledge (knowledge not determined by personal experience) to guide behavior. In this regard, the authors consider acquisition of ToM to be one of the hardest subjects of cognitive development. As a mathematical technique, Bayes’ law works very differently from projection. Neurophysiologically, there appears to be extreme locational correlation between neural signals involved in Bayesian inference in probabilistic tasks, and mentalizing in games: right Temporo-parietal Junction (rTPJ), for instance, activates in both types of tasks, and the activation appears to be causal to ensure good performance^14,15^. Game theory, a subfield of mathematics, provides an abstract environment with which to predict play in strategic environments. This theory assumes a “model” of the opponents, representing them as purely selfish agents who best-respond given conjectured actions of others in the game. Not only does game theory describe human strategic interaction well^16^; the mathematical calculations underlying its predictions correlate with neural signals in brain regions widely considered to be associated with ToM, such as rTPJ (mentioned before), or paracingulate cortex^9^. Finally, there exists emerging evidence of correlation between deficiencies in ToM and in abstract thinking in certain mental disorders such as schizophrenia^17,18^. This correlation is reflected in reduced activation of putative ToM brain regions^19^. And the deficiencies in ToM correlate with well-defined patterns of mistakes in game-theoretic computations^20^.

As such, the ability to think outside personal context is common to well-pronounced ToM^7,8^ and to avoiding DE. We thus wondered whether individuals who are talented at social-cognitive component of ToM may be able to deploy their ability to abstract to the domain of finance. Here, we ask how the transfer of this skill can be accomplished in practice. Importantly, we wanted the transfer to be subconscious, because many aspects of investments (mathematical intricacies, unusual vocabulary, unusual uncertainty) may stand in the way of conscious transfer; conversely, consciously becoming financially literate may come at the cost of overconfidence^21^.

We designed a training scheme aimed at subconsciously transferring good ToM function to investments. We performed a longitudinal (multiperiod) experiment with a four-week treatment washout period to determine its efficacy^22^. Our methodological approach adopts the key premise underlying cognitive training schemes: a specific cognitive function is exploited through a structured intervention, resulting in the intended behavioral change (see, e.g., the scheme from Chambers and collaborators^23^). To our knowledge, we are the first to propose the use of a cognitive training scheme to transfer good function from one domain (the social sphere) to another (investments).

We administered additional tests in order to confirm that the function transfer scheme worked because of the cognitive dimension of social function, as opposed to the emotional dimension. Investors with better generic social function have been shown to predict more accurately price changes in markets with insiders and to sell more timely when bubbles emerge^24–27^. In our experiment, however, participants who scored high on tests of the cognitive dimension of social function were initially no less susceptible to DE. To corroborate the hypothesized link between the ability to abstract and the success of our intervention, we investigated whether a reduction in DE was associated with reduced attention paid to personal circumstances, in particular, the price at which a financial position was acquired. We deployed eye tracking in order to trace attention to various pieces of information, including the acquisition price, that were displayed in the computer user interface^28^.

Participants were subjected to two interventions, separated by four weeks to allow for treatment washout and subsequent re-uptake^29,30^. The size of the sample was determined by means of power analysis based on Frydman and Rangel (2014)^28^, which demonstrated a small (25%) impact on DE from simply deleting information about the acquisition price. Each of the interventions consisted of four sessions: (I) a session organized along the lines of the experiment by Frydman and collaborators^31^, to measure the extent to which the participant’s investment decisions are affected by the DE; (II) a session whereby the participant (advisor) chooses investments for a person (advisee) selected by the advisor; importantly, the advisee always realizes gains (or losses) immediately after the end of an investment trial, so the advisee is never invested when the participant recommends investment; (III) a session that combines (I) and (II), whereby the advisor chooses investments for herself, as well as for the advisee (selected in the previous session) who immediately realizes gains (or losses) upon conclusion of an investment trial; (IV) a repetition of session (I), to measure the impact on DE of the training intervention. See Fig. 1B.

Sessions (II) and (III) constitute the core of our intervention. There, participants are asked to decide on an advisee’s behalf. The best decision requires them to ignore their own situation, because the advisee is never invested at the moment the advice is given, while participants may be invested (in Session III). By requiring decisions for both the advisee and for themselves, in Session (III) we nudge the participant to reflect on the relevance for his/her own investments of the action chosen for the advisee. If s/he is holding a position and the decision for the advisee is not to invest, why would the participant him/herself remain invested? If the advice is to buy, then the participant should conclude that s/he too should remain invested. Etc.

In all sessions, the investment game was the same. Participants took positions (long; short) in one share of a security called “stock” that went through good and bad regimes. In the good regime, the stock price went up the majority of the time; in the bad regime, the stock mostly went down. Regime switches occurred randomly. See Fig. 1C and Methods. Participants knew that there were regime switches, they were also aware of probabilities, and the possible magnitudes of the outcomes in each regime. In any trial, the regime had to be inferred, as participants were never told which regime the stock was in. But a sequence of mostly positive (resp. negative) price changes revealed that the stock most likely was in the good (resp. bad) regime, and hence, that one was to be “long,” which means that one should have bought (resp. “short,” i.e., selling the stock with the aim of buying back later at a higher price). At the end of each of the four sessions in the test and retest parts of the study, participants’ holdings of the stock A were liquidated, and the cash value of their position was recorded. Participants’ incentives depended on the final value of their portfolio at the end of each session.

Before trading sessions (II) and (III) of both the test and retest treatments, participants were shown photographs of 21 potential clients, among which they could select the one whom they would advise. The purpose behind the diversity of the presented photographs was to increase the chances of interpersonal attraction of the participants to the person that they selected to help^32,33^, and thereby the efficacy of the intervention. In session (IV), participants only traded for themselves, as in the session (I).

We measured DE as in Odean (1998)^34^. The measure compares the frequency with which gains are realized against the frequency of loss realizations. See Fig. 1D and Methods. DE emerges if this measure is positive: more gains than losses are realized. In fact, the Bayes-optimal policy in our investment game even implies that more losses have to be realized than gains. That is, the DE measure should be negative ($-0.73$). This extreme number assumes, however, that one can immediately switch from a long to a short position and *vice versa*, something we disallowed: a long position had to be liquidated before a short position could be taken in the subsequent trial. In prior experiments, participants rarely did better than reaching balance between realized gains and losses^31^.

We gauged ToM function using three subscales of The Awareness of Social Inference Test - Revised (TASIT-R^35^). The subscales delineate function associated with social-perceptual and social-cognitive components of ToM. The subscales are: the Emotion Evaluation Test (EET), the Social Inference-Minimal (SI-M) test, and the Social Inference-Enriched (SI-E) test. EET gauges the social-perceptual ToM, while the social inference (SI) tests measure the social-cognitive component of ToM. Different forms were used between the test (Form A) and re-test (Form B) sitting. All the tests are used in clinical settings. We refrained from using popular academic ToM tests, such as the “Reading the Mind in the Eyes” Test (RMET^36^), because they commonly measure only one component of ToM (for example, RMET measures only the social-perceptual component of ToM^4,6^). Scores on the test were used to predict DE level in a session and changes in levels across sessions and sittings.

Eye movements, and from them, eye fixations, were recorded using a table-mounted eye tracking system. For each trial, eye fixations were calculated in relation to the areas displaying the acquisition price, the market price, and the overall trading dashboard. See Methods.

To avoid deception, we referred in the instructions to the investment game as the “Disposition Game,” and described it as a game where “you will attempt to maximize profits while buying and selling a security and avoiding the Disposition Effect” (See Supplementary Information, Appendix C). Participants were not incentivized monetarily to improve DE. If asked, DE was explained as the tendency to sell too early on gains while hanging on to losses too long. Participant earnings only depended on the final value of the positions they took for their own account. No other feedback was provided besides final portfolio value in a trading session. The putative role of the investment advice to others in potentially mitigating DE was not explained.

## Results

The behavioral analyses were based on 68 participants retained from the original sample (see Supplementary Information). Of these, 52.9% were male, 45.6% were female, while one participant (1.5% of the sample) preferred not to disclose the gender. The average age of the participants was 21.22 years (SD = 1.92), ranging from 18 to 27 years. 16.2% of the participants had prior trading experience outside the academic setting, while the rest (83.8%) did not have such experience. In the analyses of eye tracking data, further five participants had to be removed due to a high amount of missing eye tracking data (we imposed a data quality threshold of 85%; see Supplementary Information).

The average participant displayed a highly significant tendency to more frequently realize gains than losses (mean DE measure = 0.12, $p < 0.001$). After Holm-Bonferroni Familywise Error (FWE) correction, DE scores overall did not increase with scores on any of the six ToM tests, regardless of whether they concerned the cognitive or perceptual dimension ($p > 0.10$). When averaging scores across forms A and B of the SI-E test, participants with above-median average scores realized gains with a frequency that was 14.8% percentage points higher than the frequency for losses (SD = 0.31).

**Figure 2:**
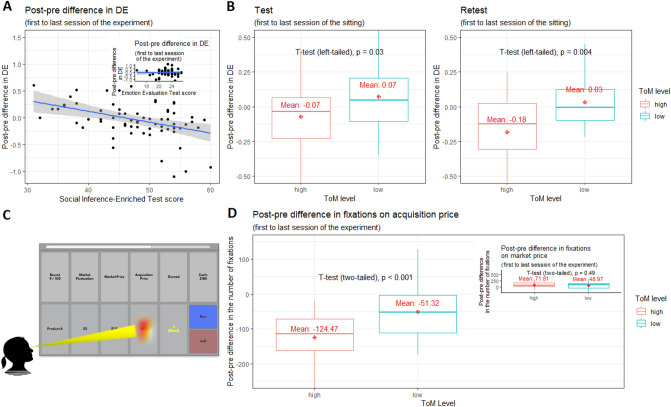
(**A**) The results of OLS regression analyses where Social Inference-Enriched (SI-E) test score (and Emotion Evaluation Test (EET) score in the inset) is used as a predictor of the change in disposition effect, calculated as post-pre difference in disposition effect, reveal that Disposition Effect (DE) decreases significantly from session I of first sitting to session IV of second sitting, as a function of score on the Social Inference-Enriched (SI-E) test score. (Inset: For comparison, DE does not change as a function of the Emotion Evaluation Test (EET) score). (**B**) Boxplots of changes in DE for two cohorts based on median split of average SI-E scores (forms A and B), during test sitting (Left) and retest sitting (Right). (**C**) Display of game interface, featuring Round, Market Fluctuation since previous round, Market Price in current round, Acquisition Price, indication whether stock is Owned ($+1$) or sold short ($-1$), Cash still available in round, and two choice panels (Buy or Sell; if position is long (+1), Buy is unavailable and hence grayed out; if position is short (-1), Sell is unavailable and hence grayed out). Here, participant’s eye fixation is on Acquisition Price panel. (**D**) Boxplots of post-pre differences in number of fixations on acquisition price panel relative to total number of fixations, from the last session (IV) to the first session (I) of the experiment, stratified by social-cognitive ToM score (Inset: for comparison, post-pre differences in fixations on market price panel, from last (IV) to first session (I) of the experiment).

Overall, using univariate OLS regression analyses, scores on tests of the social-cognitive component of ToM, whether taken during the test or re-test sitting, predicted significant reduction in DE from the first session in the test sitting (I) to the last session in the re-test sitting (IV) (Fig. 2A) [SI-M, Form A: $F(1,66) = 4.13$, $p = 0.046$, $f^2=0.004$ (small effect); SI-M, Form B: $F(1,66) = 8.21$, $p = 0.006$, $f^2=0.012$ (small effect); SI-E, Form A: $F(1,66) = 6.22$, $p = 0.015$, $f^2=0.008$ (small effect); SI-E, Form B: $F(1,66) = 11.93$, $p < 0.001$, $f^2=0.023$ (medium effect)]. Scores on tests of the social-perceptual component of ToM (EET) did not produce significant effects (Fig. 2A, inset) [$p > 0.10$, both Forms]. Holm-Bonferroni FWE correction at $p = 0.05$ confirms the significance of the effect of social-cognitive ToM on reduction in DE through the training intervention, but only for the Form B versions of the SI-M and SI-M tests (taken during the re-test sitting); the Form A versions (taken at the initial test sitting) were marginally insignificant. One point increase in the scores produced a reduction in DE of between 1 and 2 percentage points (depending on the test and the form used).

Averaging SI-E scores across forms A and B of an individual and performing a median split across participants^37^ produced a large improvement (drop) in the mean DE scores, of 85.4%, in the group above the median. A paired-samples *t* test indicated a significance of $$p = 0.042$$ when comparing the scores obtained in the last to first session of the experiment [left-tailed test of whether average DE score change of high-ToM group is zero; $$t(34) = -1.79$$, $$d = -0.38$$ (small effect)] . In contrast, the group below the median actually increased its mean DE score (from 0.09 to 0.14), and hence performed worse, though this increase was not significant ($$p > 0.10$$). Further, independent-samples *t* tests compared post-pre difference in DE between high- and low-ToM groups from first to last session of the sitting (left-tailed test of whether average DE score change of high-ToM group is equal to that of low-ToM group). Within-sitting effects on post-pre difference in DE scores are displayed in Fig. 2B. In both sittings the difference in change of DE between high- and low-ToM groups was significant [left-tailed tests; test sitting: $$t(66) = -1.91$$, $$p = 0.03$$, $$d = -0.48$$ (small effect); retest sitting: $$t(66) = -2.73$$, $$p = 0.004$$, $$d = -0.69$$ (medium effect)]. Further, analysis of covariance (ANCOVA), conducted to eliminate the biasing effects of individual differences in the baseline, indicated that, after accounting for the effects of DE level measured during the first session of the test sitting (I), the effect of ToM group on post-pre difference in DE scores (first to last session of the sitting) in the test sitting was not significant [$$F(1,65)=2.70$$, $$p = 0.105$$, partial $$\eta ^{2} = 0.04$$]. However, in the retest sitting ANCOVAs indicated a significant effect of ToM group on post-pre difference in DE, either when accounting for the effects of DE level measured during the first session of the test sitting (I) [$$F(1,65)=6.67$$, $$p = 0.012$$, partial $$\eta ^{2} = 0.09$$ (medium effect)], or when accounting for the effects of DE level measured during the first session of the retest sitting (III) [$$F(1,65)=4.72$$, $$p= $$0.033,  partial $$\eta ^{2} = 0.07$$ (medium effect)].

There was marginal evidence (FWE $p < 0.10$) of resurgence of DE between the last session (IV) in the test sitting and the first session (I) in the retest sitting as a function of score on the social-cognitive dimension of the ToM tests, but ultimately the strong and highly significant training effect for high scores on those tests (SI-M and SI-E tests) in the re-test sitting more than offset this reduction. Even without correction for multiple hypothesis testing, scores on tests of social-perceptual ToM (EET tests) never produced significant ($p > 0.05$) impact on DE through training. A comprehensive analysis of levels and changes of DE across all stages of the training is provided in the Supplementary Information.

We then investigated whether successful trainees stopped paying attention to the cue that was key to DE, namely, the price at which they acquired a position. Without attending to this price, gains and losses were undefined, and DE could have emerged only by accident (it could of course have been the case that participants always remembered the acquisition price, but this should be considered implausible given the amount of data that the participant would have had to retain). We measured selective aspects of participants’ attention using eye gaze^38,39^: if participant’s eyes were oriented towards an object (e.g., the purchase price), we assumed that she was paying more attention to the object than to others on the screen. Following Holmqvist e.a. (2011)^40^, total fixation count on the trading dashboard was used as the base measure of eye gaze fixations, and fixations on an area of interest (AOI) was counted against this base. As area of interest, we took the cell in the trading window displaying the acquisition price (see Fig. 2C). Figure 2D shows boxplots of the change in average number of fixations on the acquisition price panel relative to total number of fixations from first to last session of the experiment, stratified by social-cognitive ToM score (average of SI-E scores [forms A and B] per individual, median split across individuals). An independent-samples *t* test of the differences in the means across groups produced a highly significant *p* value and a large effect size [two-sided test of whether decrease in fixation frequency in the high group is equal to that of the low group; $t(61) = 4.56$, $p < 0.001$, $d = 1.15$].

In way of contrast, change in fixations on the current market price relative to total number of fixations from first to last session of the experiment did not differ significantly ($p > 0.10$) across ToM groups (Fig.  2D, inset). The current market price is a relevant piece of information regardless of whether choices display DE. Participants who chose rationally needed to determine whether the current market price is beneficial relative to future expected prices. Participants who displayed DE chose based on a comparison between the current market price and the market price at which they established their position.

Finally, we conducted post hoc analyses to investigate the role of the trading experience on the proneness to DE across experimental sessions. Multiple regression analyses with ToM score and trading experience as predictor variables revealed no significant effect of trading experience on change in DE, calculated as post-pre DE. When considering the effect of trading experience on DE level, we found that participants with trading experience had a significantly ($p < 0.05$) higher level of DE in session (IV) of the first sitting and in sessions (I) and (III) of the second sitting, while in other sessions the effect of trading experience on DE was not significant. (Significance levels are not adjusted for multiple hypotheses testing, so should be interpreted with caution.) Controlling for trading experience while testing the effect of social-perceptive and social-cognitive aspects of ToM on the level of DE and post-pre change in DE generally resulted in more significant findings for the tests already reported above as significant. However, this did not add any extra insights.

## Discussion

We proposed and tested a novel cognitive training scheme meant to transfer the human capacity to abstract from one’s own history when forming a theory of what others may be thinking, to avoiding an investment bias which has its roots in false reference to personal history. Our longitudinal test-retest experiment demonstrated an increase in efficacy of the scheme as a function of the score on tests for the cognitive dimension of ToM. Scores on tests for the social-perceptual component of ToM did not correlate with DE score improvement, further corroborating our hypothesis that the scheme worked because the abstraction component of ToM is what matters for the transfer to be successful.

Participants who scored above the median on our tests of social-cognitive component of ToM reduced their average DE from about 15% to about 2%, a reduction of more than 85%. The above-median participants thus ended up, on average, balancing the frequency with which gains and losses were realized. A significant fraction managed even to realize more losses than gains, consistent with the Bayes-optimal policy: 1st quartile DE = -0.04. Participants below the median scored worse on DE after training (DE scores doubled, from 0.08 to 0.14 on average) but this difference was statistically insignificant ($p > 0.10$). Consistent with the putative role of ToM we found that our training scheme produces significantly higher DE improvements the higher one scores on the tests of social-cognitive component of ToM, but not the social-perceptual component of ToM.

Our intervention builds on the role that social-cognitive component of ToM plays in supporting a higher-order cognitive skill. We disregard interaction between DE and social environment. Such interaction has been documented: the desire to manage self-image, for instance, may exacerbate DE when one’s actions are being scrutinized by others^41^. We would argue that our training scheme is even more relevant in such circumstances, since it would require one not only to abstract from personal investment history, but also to abstract from being tracked by others.

Our experiment does not necessarily shed any light on the causes of DE, only that it can be attenuated by transfer of ToM function, and that this attenuation increases with social-cognitive component of ToM function. Other known, albeit controversial, ways to reduce DE are financial education^42,43^ and investment experience^44,45^. The literature has also considered the role of contextual cues. For example, using a between-subject laboratory experiment^28^, tests whether reducing the saliency of a stock’s purchase price by not displaying it on the trading dashboard would result in a reduction of the disposition effect. They recorded a drop of 25% in DE, compared to our 85%. Likewise, a natural experiment involving salience of the purchase price increases DE by 17%^46^. There are doubts, however, that manipulating display of acquisition prices would work in practice: traders and investors may expect to see acquisition prices; deletion thereof may draw attention, and hence produce the opposite effect from intended.

Our ToM function transfer training should work in other contexts as well. Most closely related is the *sunk cost fallacy*^47^, whereby managers, politicians and administrators stick to an investment (e.g., an inner-city metro transit system construction) even if nobody would want to take it over for a positive price, merely because they have already spent resources on it in the past. More generally, good ToM function should be able to help overcome the ubiquitous use of faulty (and often manipulable!) reference points in decision-making^48^ when these reference points relate to one’s personal circumstance.

There is a close relationship between Bayesian inference and the social-cognitive aspect of ToM. Both concern the determination of hidden (latent) variables: intentions of others for ToM; causes behind observables in the case of Bayesian inference. It is therefore not surprising that search for the neurobiological foundations of *either* of them produced results that are relevant for *both*. Regions such as paracingulate cortex or temporo-parietal junction appear to be engaged in both tasks^49^ and attempts to distinguish their involvement have rarely been met with success^14^. It could be that search for differentiation is futile, for indeed, besides context, one could ask: is there any difference between discerning, through actions, intentions of an opponent in a strategic game^9^, and discerning, through accumulated rewards in a probabilistic task, whether contingencies have reversed^50^? The present study underscores how close the two are, in showing that good function in one context can enhance function in another one.

The latter raises an important question: can we accomplish the opposite transfer? Can we transfer abstraction in humans who exhibit no DE in investments yet are deficient in the social-cognitive component of ToM? Especially in the context of certain types of neurodevelopmental disorders that involve selective impairments in social-cognitive aspects of ToM (for example, Asperger syndrome^51,52^), can one convert, through training of the kind advocated here, successful investment skill into improved social cognition? If so, novel therapies for people with such neurodevelopmental disorders could be envisaged. We leave this for future research.

## Methods

We ran a longitudinal pre-post intervention design^22^. Ethics approval was obtained from Monash University, where the research study was conducted; Ethics Number CF16.346 - 2016000160. All methods were carried out in accordance with relevant guidelines and regulations. Informed consent was obtained from all participants prior in the experiment. All participants were at least 18 years old and were recruited through a student research participation pool at Monash Business School, Australia. See Supplementary Information for details.

To test for potential washout of the treatment effect, the experimental treatments were administered twice (test and retest sittings) within an interval of four weeks. The disposition effect (DE) was measured at the beginning and end of each sitting.

In the statistical analysis of the results, we employed univariate OLS regression analysis to quantify the magnitude and significance of the effect of ToM test scores on DE. We chose the first set of dependent variables (DVs) to be based on an individual measure of the disposition effect as operationalized in Odean’s article^34^ and utilized in numerous follow-up studies^28,31,53,54^. This measure was calculated as the difference between the Proportion of Gains Realized (PGR) and Proportion of Losses Realized (PLR). PGR (PLR) was calculated as a ratio between the number of realized gains (losses) and the sum of the number of realized gains (losses) plus the number of paper gains (losses). *Paper* gains/losses occurred when a participant decided not to divest.

The second set of DVs was based on the *difference* between individual disposition effect scores obtained across trading sessions. They were meant to provide a measure of learning and improvement.

The third set of DVs was associated with the degree of a participant’s attention to the acquisition price compared to overall attention paid to the trading dashboard. These measures captured the proportion of eye fixations on the acquisition price relative to eye fixations on the overall dashboard.

Independent variables (IVs) in the regressions were based on ToM, as assessed using three subscales of the Awareness of Social Inference Test - Revised (TASIT-R^35^), which delineated and measured social-perceptual and social-cognitive components of ToM. See main text for subscales. With these IVs, we investigated whether the level of social cognition (as per the social inference subscales of the TASIT-R tests) versus the level of emotional cognition (as per the emotional inference subscale of the TASIT-R tests) were associated with (i) the level of the DE measure, (ii) the reduction of DE during the experimental interventions, (iii) reduction in eye fixations on the item in the user interface that drives DE, namely, the purchase price.

Throughout, we made Holm-Bonferroni adjustments to significance levels, to account for multiple hypothesis testing, and hence, to correct for Family Wise Error (FWE). For instance, when studying the impact of scores of the six possible social function tests on DE, we accounted for the six resulting tests as follows. We ordered test *p* values from small ($k=1$) to large ($k=6$); the *k*th test value was deemed to be significant at the level $\alpha$ if $p(k) \le \alpha /(m+1-k)$ where *m* was the number of hypotheses to be tested; here: $m=6$. If $\alpha = 0.05$ then the smallest *p* would have to be $\approx 0.008$ for the corresponding test (i.e., the test with smallest *p* value) to reject. For the next test to reject, its *p* value could at most be 0.01. Etc.

As an alternative to regressions that relied less on parametric assumptions (e.g. linearity; homoscedastic errors), we also performed a median-split based on the ToM test scores and compared average DE score levels and changes across trading sessions. Paired-samples and individual-samples *t*-tests were performed on levels and changes. When reporting *p* values for one-sided tests, we focused on the tail of the distribution where the distribution of the statistic under the alternative hypothesis overlapped most with that under the null hypothesis. We also ran analysis of covariance (ANCOVA), conducted to eliminate the biasing effects of individual differences in the baseline.

Throughout, effect sizes were estimated using Cohen’s *d* and $f^2$ statistics, as well as partial $\eta ^2$ statistics. Statistical results were inspected graphically using scatter plots and boxplots.

The experimental task closely followed that of Frydman and collaborators^31^, which itself was based on the earlier stock trading task developed by Weber and Camerer^55^. Participants were given the opportunity to trade one stock, named stock A. Each participant was allowed to hold a maximum of one(‘1’) share and a minimum of minus one (‘-1’) shares (negative positions corresponded to short-selling). Participants bought or sold at the current-trial market price.

The price path of stock A was governed by a two-state Markov chain with a good state and a bad state. Suppose that, in trial *t*, $t = 1, 2, ..., 100$, there was a price update for stock A. If stock A was in the good state at that time, its price increased with probability 0.55 and decreased with probability 0.45. Conversely, if it was in the bad state at that time, its price increased with probability 0.45 and decreased with probability 0.55. The magnitude of the price change was drawn uniformly from $\{5, 10, 15\}$, independently of the direction of the price change. The state of each stock evolved over time in the following way. Before trial 1, stock A is randomly assigned a state. With the price update in trial $t > 1$, the state of stock A in the trial remained the same as in trial $t - 1$ with probability 0.8, but switched with probability 0.2.

The computer graphical user interface was written in Unity3D (http://unity.com). It gave participants access to their holdings, current price in the trial, acquisition price (if invested), and market fluctuation (price change since last trial). When trading on behalf of another, the graphical user interface was identical, except that the acquisition price did not apply (since the advisee never cashes in every round), and a picture of the (chosen) advisee was added.

Participants were paid a sign-up reward plus their earnings from trading for their personal account. In the second trading session of both the test and retest sittings, participants were asked to recommend purchases or (short-) sales to a client. Details of the client selection protocol can be found in the Supplementary Information. After selecting a client (an advisee) from among 21 photographs (see Supplementary Information), participants traded on behalf of their advisee. Importantly, the advisee never held on to investments for more than one trial. That is, gains and losses were realized immediately. As a result, there could not be a DE.

During game play, eye movements were recorded using a table-mounted eye tracking system (Tobii TX300; Tobii, Stockholm, Sweden) with a temporal resolution of 300 Hertz and a screen resolution of 1920 x 1080 pixels. Eye fixations were computed using the velocity-based I-VT algorithm^56^. Frequencies of fixations on the acquisition price and on the market price were compared based on a median split of the data based on ToM test scores. Two-tailed independent-samples *t*-tests were used to assess significance of differences in the two types of fixations across groups. Cohen’s *d* statistic measured effect size.

## Supplementary Information

Supplementary Information.
