# Supplementary material for: Transferring cognitive talent across domains to reduce the disposition effect in investment
Source: Sci Rep. 2021 Nov 29;11:23068. doi: 10.1038/s41598-021-02596-2 (PMC8630220; doi:10.1038/s41598-021-02596-2)
Supplement: Supplementary file 1 — Supplementary Information. [file 41598_2021_2596_MOESM1_ESM.pdf]

Transferring Cognitive Talent Across Domains

to Reduce the Disposition Effect in Investment

K. Rotaru, P. Kalev, N. Yadav, P. Bossaerts

SUPPLEMENTARY INFORMATION

1. Experimental Design in Detail

1.1. Summary of procedures

Our training protocol is organized as follows. There are two interventions, separated by four weeks to allow for treatment washout and subsequent re-uptake. The investment game is borrowed from Frydman, Barberis, Camerer, Bossaerts and Rangel[1] but is limited to one security rather than three. In addition, participants can change investments after each trial. As such, it is easier to implement the Bayes-optimal strategy.

The situation is simple. Participants take positions (long; short) in one share of a stock that goes through good and bad "regimes." In the good regime, the stock price goes up the majority of the time; in the bad regime, the stock mostly goes down. Regime switches happen randomly. Participants know that there are regime switches, which occur randomly over time, as well as the possible magnitudes of the outcomes in each regime.

We measure the disposition effect (DE) as in Odean's seminal article[2]. The measure penalizes for paper losses and realized gains, since the Bayes-optimal policy is to stay invested upon gains (suggesting that the stock is in the good regime), while divesting, and even shorting, upon losses (which indicate that the stock is in the bad regime).

1.2. Participants

Seventy six undergraduate and postgraduate students enrolled in Business and Commerce programs in Monash University, Australia voluntarily participated in this study. Participants aged 18 years old or above were recruited through a student research participation pool at Monash Business School. The recruitment and management of the student research participation pool was approved by Monash Human Research Ethics Committee (Project number 9110). Eight participants (10.53% of the original

sample) were removed from the sample as they did not attend the retest laboratory sessions, so the behavioral analyses were based on 68 participants. In the analyses of eye tracking data, further five participants had to be removed due to a high amount of missing eye tracking data (we imposed a data quality threshold of 85%; see Appendix A). The exclusion rate due to missing eye tracking data reported in our study is in line with prior eye tracking literature[3,4].

The choice of sample size was based on the study of Frydman and Rangel[5]. By reducing the visual saliency of the purchase price Frydman and Rangel found a significant reduction in the DE with 58 participants. As such, a sample size of 58 appears to generate sufficient power to evaluate the efficacy of interventions. Consequently, we aimed at the same number of participants. We ended up with more valid records (68 instead of 58). We too found a significant effect. Importantly, our effect size was far bigger.

1.3. Intervention Design

We considered a longitudinal pre-post intervention design (as in the design by Chambers, Lo and Allen[6]). This meant that participants had to make a sequence of decisions across multiple trading sessions. We aimed to investigate the effectiveness of the proposed ToM-based training scheme to reduce the DE. To test for potential washout of the treatment effect, the experimental treatments were administered twice (test and retest sittings) within an interval of four weeks. DE was measured at the beginning and end of each sitting.

In the statistical analysis of the results, we chose the first set of *dependent variables (DVs)* to be based on an individual measure of the DE, as operationalized in Odean's work[2] and utilized elsewhere[1,5,7,8]. This measure is calculated as the difference between the Proportion of Gains Realized and Proportion of Losses Realized as outlined in the Methods of the article.

The second set of *DVs* was based on the *difference* between individual DE scores obtained for each of the trading sessions. They were meant to provide a measure of learning and improvement.

The third set of *DVs* was associated with the degree of a participant's attention to the acquisition price compared to overall attention paid to the trading dashboard. These measures capture the proportion of eye fixations on the acquisition price relative to eye fixations on the overall dashboard.

Independent variables (IVs) were based on ToM, as assessed using three subscales of the Awareness of Social Inference Test - Revised (TASIT-R[9]), which delineate and measure social-perceptual and social-cognitive components of ToM[10-11]. As explained in the main text, the subscales are: the Emotion Evaluation Test (EET), the Social Inference–Minimal (SI-M) test, and the Social Inference–Enriched (SI-E) test. With these IVs, we investigated whether social-cognitive component of ToM (as per SI-M and SI-E tests) versus social-perceptual component of ToM (as per EET test) were associated with (i) the *level* of the DE, and (ii) the *reduction* of the DE before and after the experimental interventions. We used regression analysis and median-splits to determine statistical significance and size effect. See Methods.

1.4. Stock Trading Task

The experiment was based on the stock trading task introduced by Weber and Camerer[12] and followed the experimental design by Frydman, Barberis, Camerer, Bossaerts and Rangel[1] and Frydman and Rangel[5]. The experiment was based on four sessions separated by a two-minute break. Each session lasted for an average of 17 minutes and consists of 100 trials. Before each session, the participant was given $50 in experimental currency. The participant was then asked to buy one share of stock. The initial share price for stock A was $100. Once a stock was purchased, the acquisition price was updated on the trading dashboard (see Figure 1). Cash positions could become negative. No interest was charged on negative cash positions.

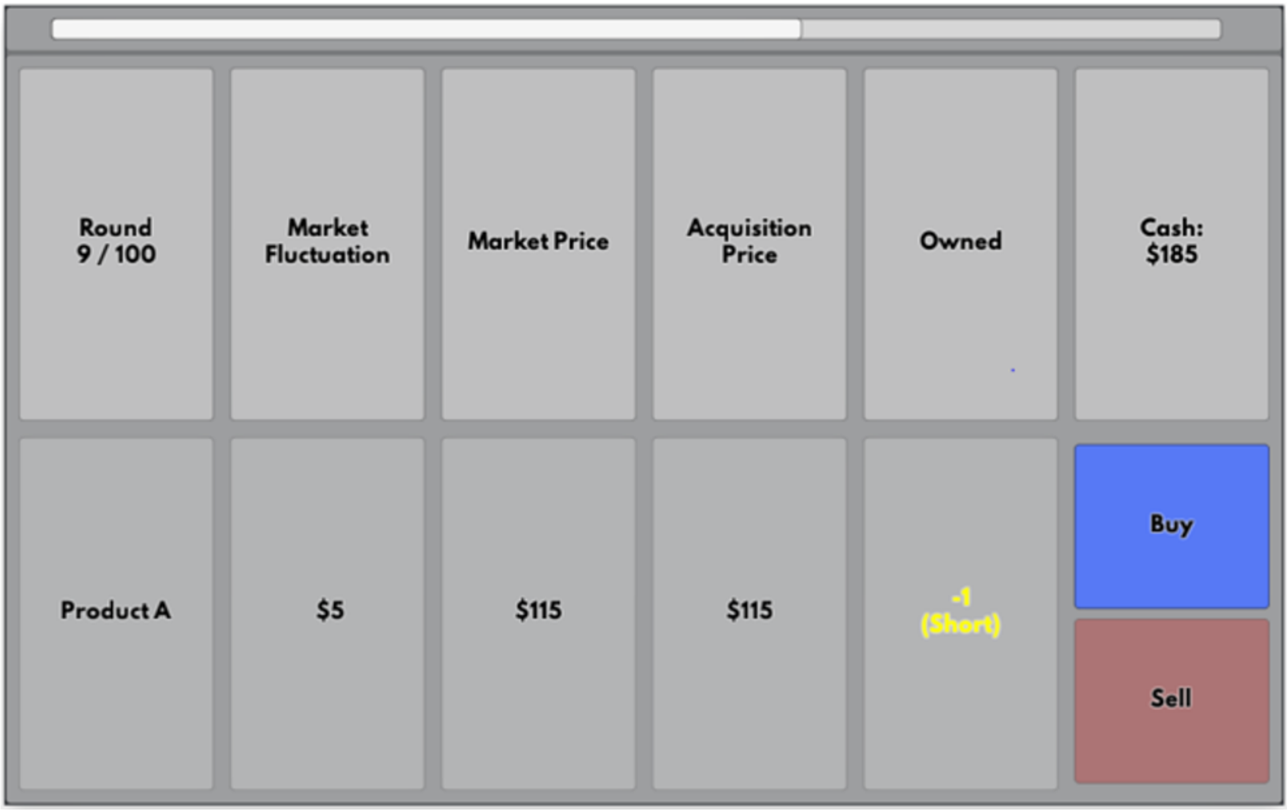

Figure 1. Trading dashboard.
Version of the research instrument where participants traded for their own account.

In line with the design of Frydman and collaborators[1], the state of each stock evolved over time in the following way: if $s_{i,t}$ ∈ {*good, bad*} was the state of the *i*-th stock in trial *t*, then the state switched as follows:

	$s_{i,t}$ = ***good***	$s_{i,t}$ = ***bad***
$s_{i,t-1}$ = ***good***	0.8	0.2
$s_{i,t-1}$ = ***bad***	0.2	0.8

It was expected that participants would infer the states of the stocks from the observed price paths. The states of the stocks were never revealed to them. The same set of realized states and prices

was used for all participants, to facilitate comparability. The optimal trading strategy for a risk-neutral Bayesian investor whose objective is to maximize the expected value of her take-home earnings is formalized in the aforementioned study by Frydman and collaborators[1].

Financial incentives: At the end of each of the four sessions in the test and retest parts of the study, participants' holdings of the stock A were liquidated, and the cash value of their position was recorded. Participants' incentives depended on the final value of their portfolio at the end of each session. Specifically, if the total value of a participant's cash and risky asset holdings at the end of session 1 was X_1, the total value of her cash and risky asset holdings at the end of session 2 was X_2, at the end of session 3 was X_3, and at the end of session 4 was X_4 (in experimental currency), then her take-home pay in actual dollars was $10 + (X_1 + X_2 + X_3 + X_4)/48$. The structure of the financial incentives was communicated to the participants before the start of the experiment (see Appendix C).

1.5. ToM-Based Intervention Using the Nencki Affective Picture System (NAPS)

In the second trading session of both the test and retest treatments, participants were asked to recommend purchases or (short-) sales to a client. Participants were shown photographs of 21 clients (see Figure 2), among which they could select one whom they would advise. The second trading session was a core component of the suggested intervention policy whereby we attempted to transfer ToM function to personal investment decisions. The efficacy of this policy was dependent upon participants' willingness to help the potential investor, who never currently owned the asset at hand, by deciding (to purchases or to (short-)sell) on their behalf. The operationalization of this intervention policy with the use of diverse pictures drew on the evidence that people often infer traits from the facial appearance of other people[13-14]. The literature also suggests that liked people receive more help than do disliked people[15], while it has been experimentally demonstrated that even a 100 ms exposure to a human face results in significant attributions of traits such as attractiveness and trust[14].

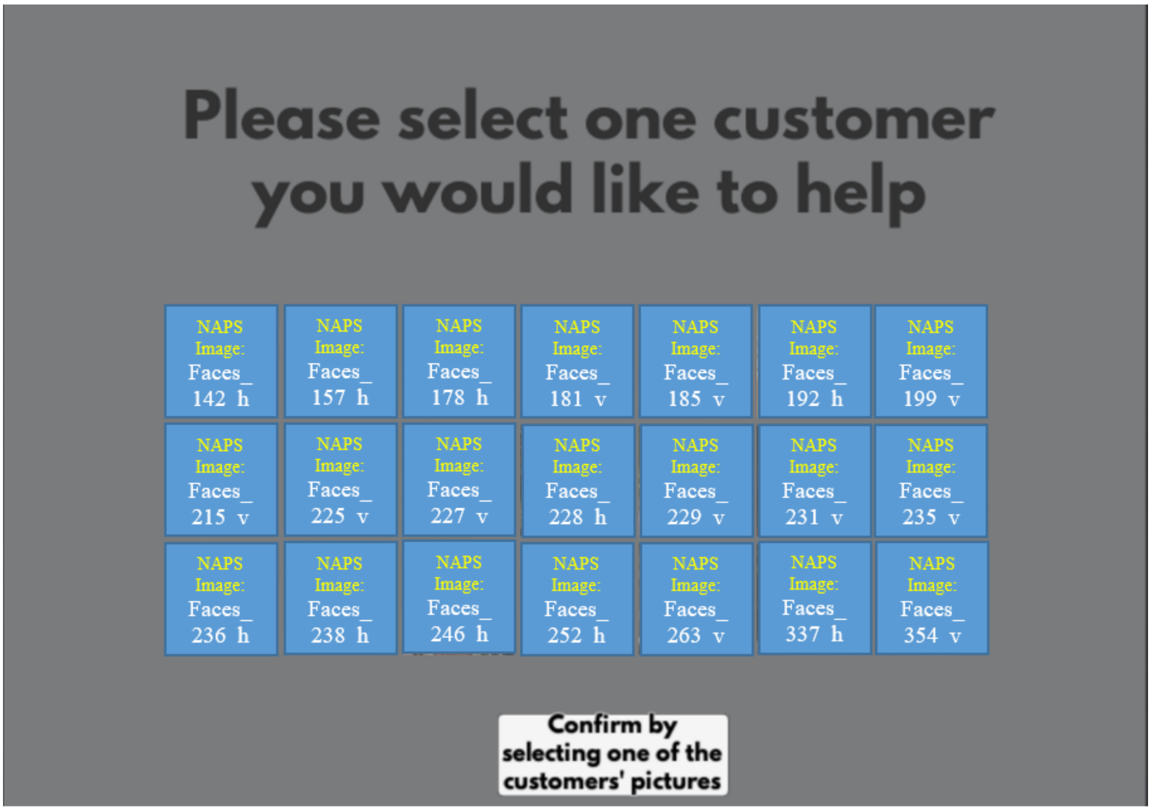

Figure 2. Advisee selection screen.

Note: Due to copyright restrictions, the original images that were used in the experiment cannot be reproduced here. However, we report the image codes/titles, as per Nencki Affective Picture System (NAPS)

The photographs were selected from the Nencki Affective Picture System (NAPS [16-17]). The approval to use NAPS images was obtained from the Laboratory of Brain Imaging (LOBI), Neurobiology Center, The Nencki Institute of Experimental Biology on 2.15.2017. The photographs were chosen to ensure diversity, as to age, gender, and ethnic background. The purpose behind the diversity of the presented photographs was to increase the chances of interpersonal attraction of the participants to the person that they selected to help. The suggested intervention policy was in line with recently proposed 'people as means approach to interpersonal relationships'[18], according to which "people serve as means to goals—helping other people to reach their goals in a variety of ways, such as by contributing their time; lending their knowledge, skills, and resources" (p. 373). In case of the present experiment, participants were invited to serve as means to goals of the selected clients (see Instructions in Appendix C). Sufficiently high levels of valence, arousal, and approach/avoidance,

as reported in NAPS Ratings (ibid.), were additional inclusion criteria for the images used in this study.

The measures of valence, approach/avoidance, and arousal in relation to the photographs used in this study were obtained based on the reports of 204 healthy volunteers whose demographic characteristics (119 women, 85 men; mean age = 23.9 years, SD = 3.4) were similar to those of the participants of the current study. The ratings were measured using three 9-point Likert scales. On the valence scale, participants were asked to complete the sentence, "You are judging this image as ..." (from 1 = 'very negative' to 9 = 'very positive', with 5 = 'neutral'). Next, participants judged motivational direction by completing the sentence, "My reaction to this image is ..." (from 1 = 'to avoid' to 9 = 'to approach', with 5 = 'neutral'). Finally, participants judged the degree of arousal elicited by pictures with the introductory sentence, "Confronted with this image, you are feeling: …" (from 1 = 'relaxed' to 9 = 'aroused', with 5 = 'neutral/ambivalent'). The mean ratings of the 21 photographs retained for our training sessions are as follows: valence (M = 6.23, SD = 1.31), approach/avoidance (M = 5.96, SD = 1.24), arousal (M = 4.62, SD = 1.46).

After selecting a client (an advisee), participants traded on behalf of their advisee using the trading dashboard shown in Figure 3. One distinct characteristic of this trading session is that the advisee never held on to investments for more than one trial. For this reason, DE in this trading session was not calculated; the trading session functioned merely as part of the ToM-based experimental intervention.

In the subsequent trading session, participants traded both for themselves and on behalf of the advisee (see Figure 4). Participants could choose trades for themselves ("Your A") *separately from those* for the advisee ("Other's A"). Market fluctuations for stocks "Your A" and "Other's A" were, however, perfectly correlated. As shown in Figure 4, the (potentially different) cash positions of the participant as well as of the advisee were displayed separately. In the acquisition price cells, the prices were displayed at which the stock was purchased for oneself (the advisor) and/or for the chosen "Other" (the advisee). But since the advisee was never invested for more than one trial, the acquisition

price for the advisee was always reset to “Not Available” – indicated with a dash. At the end of the session, DE was calculated and reported only for the trades placed by the advisor.

A fourth session followed, which repeated the first session, where participants traded for themselves only (see Figure 1).

The same trading sessions were administered in the retest treatment four weeks after the original test experiment.

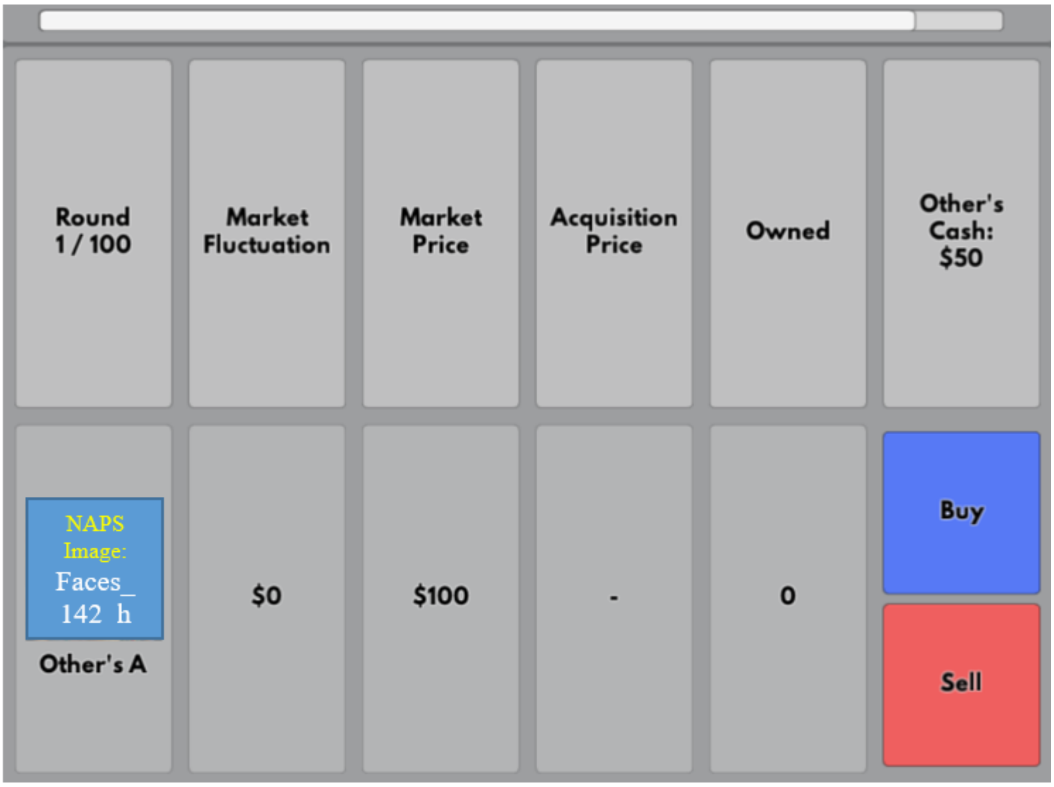

Figure 3. Trading dashboard in Session 2.
Here, participants trade on behalf of their advisee (“Other”).

Note: Due to copyright restrictions, the original image that was used in the experiment cannot be reproduced here. However, as an example, we report the image code/title, as per Nencki Affective Picture System (NAPS)

1.6. Clinical Tests of Social Cognition

We administered the Awareness of Social Inference Tests - Revised (TASIT-R).[9] Importantly, the subscales of the TASIT-R test allowed us to separate the dimensions of ToM associated with its social-cognitive and social-perceptual dimensions.

Test subscales were:

1. The Emotion Evaluation Test (EET);
2. The Social Inference–Minimal (SI-M) test; and
3. The Social Inference–Enriched (SI-E) test.

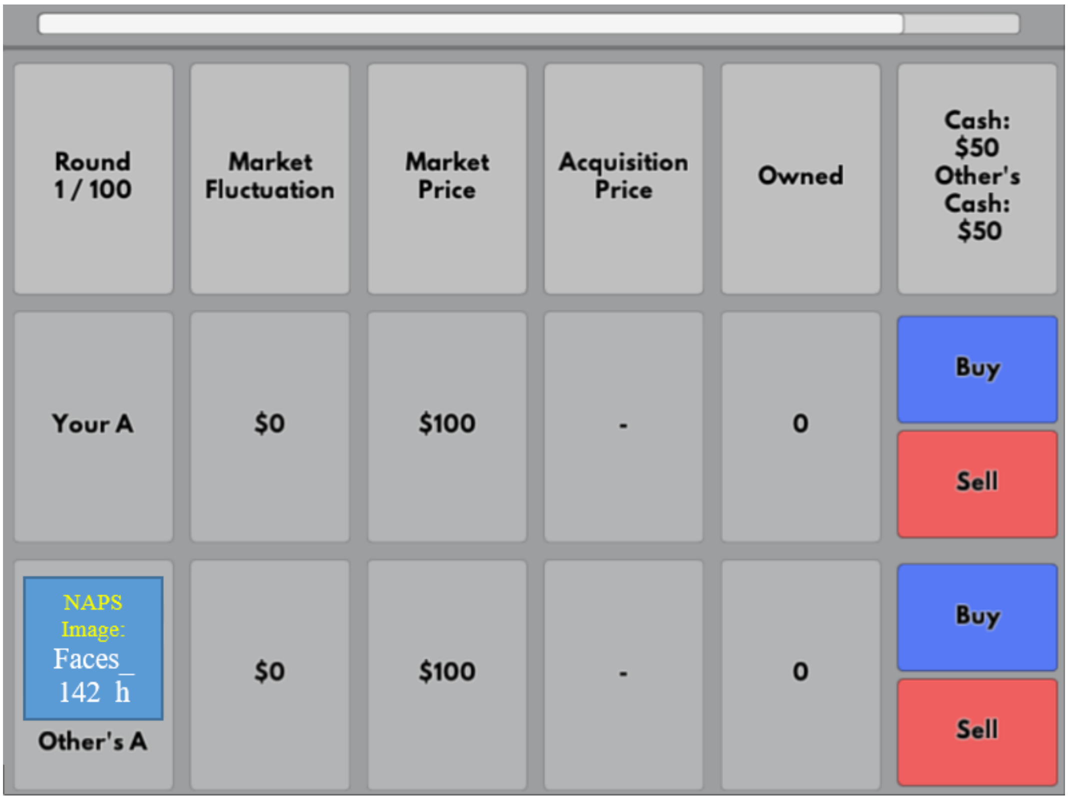

Figure 4. Trading dashboard in Session 3

Note: Due to copyright restrictions, the original image that was used in the experiment cannot be reproduced here. However, as an example, we report the image code/title, as per Nencki Affective Picture System (NAPS)

EET focuses on socio-perceptual component of ToM, while SI-M and SI-E focus on social-cognitive component of ToM. Thus, in line with the componential view of ToM [10-11], results for each of the three TASIT-R tests relate to distinct components of ToM. All three subscales in the TASIT-R test have test and retest versions (referred to as 'form A' and 'form B'), which allowed us to calibrate the test and retest treatments in our study.

1.7. Demographic Questionnaire

An online demographic questionnaire was used to record the age, gender of participants, and their trading experience.

1.8. Psychophysiological Apparatus

Eye movements were recorded using a table-mounted eye tracking system (Tobii TX300[19]) with a temporal resolution of 300 Hertz and a screen resolution of 1920 x 1080 pixels (see Appendix A for more complete technical details associated with the eye tracking technology used). At the average viewing distance of 65 cm from the screen (range: 50-80 cm), binocular accuracy of the eye tracking system was 0.4 degrees and precision was 0.14 degrees. For more detailed product specifications, please refer to Appendix A and the product description on the manufacturer's website: http://www.tobiipro.com/product-listing/tobii-pro-tx300/ Eye fixations were computed using the velocity-based I-VT algorithm[20]. For each trial, the eye tracking measures were calculated in relation to the selected areas of interest (AOIs) displaying the acquisition price panel, and the overall trading dashboard (see Appendices A and B).

The experiment was conducted in light-controlled dimly lit sound-proof booths. Participants sat on height-adjustable chairs with their head supported by a height-adjustable ophthalmological chin rest. At the beginning of the experiment, the eye tracker was calibrated using a nine-point fixation technique, which is the most rigorous calibration technique for the device used. This calibration adjusts for participants' individual differences in eye characteristics and participants' seating position.

1.9. Miscellaneous Protocol Details

An explanatory statement was presented before administering the main experimental task. Participants read the explanatory statement and signed the consent form, thereby approving the use of the de-identified data collected in this study.

The experiment was administered in the experimental laboratory (Monash Business Behavioural Laboratory) of the Monash Business School. Test and retest treatments of the study followed the same protocol. Upon signing the consent form, participants accessed the online stock trading task using a web browser link administered via Tobii Studio (version 3.4.5) software on the Tobii TX300 eye tracking systems. A maximum of six participants were trained and tested simultaneously, in separate booths with Tobii TX300 eye tracking systems. Thus, participants could not observe what other participants decided.

The remainder of the protocol was as follows. First, participants read the instructions of the stock trading game (see Appendix C) supplemented with relevant screenshots and a concise explanation of the trading dashboard. While familiarizing themselves with the rules of the game, participants could ask clarifying questions. Next, participants were administered an introductory trading session, in which they were given thirty 10-second experimental trials where they traded for themselves. Feedback in the form of the DE score and the amount (in experimental currency) earned was provided on the computer screen at the end of this introductory session. This was the only session in which the participants received the feedback in terms of a DE score. The introductory trading session took approximately five minutes to complete. This was then followed by the four main sessions in the test sitting.

The first session tested participant's susceptibility to the DE, as per the description provided in the Materials and Apparatus section (see Figure 1). In the second trading session, the participant (the advisor) chose an advisee (Figure 2) and traded on her behalf (Figure 3). The advisor was awarded a fraction of the earnings (25%) of the advisee. In the third trading session, the advisor traded both for his own account and on behalf of the advisee (Figure 4). As in Session 2, the advisee never held on to investments for more than one trial. That is, gains and losses were realized immediately, thus not allowing the DE to actualize. Compensation was based on the final value of one's own account plus a percentage of the earnings of the advisee. In the fourth session, the first session was repeated: participants traded for themselves only.

Each trading session took approximately 17 minutes to complete. Apart from their cash position, the participants did not receive any other form of feedback after the completion of a trading session.

Finally, participants were administered three subscales of the TASIT-R test (form A) in the following order: EET, SI-M, and SI-E. The completion of all three TASIT-R tests took approximately 45 minutes. Participants were also asked to complete a short demographic questionnaire, which took approximately one minute of their time.

Four weeks later, the retest treatment of the study was administered in the second sitting, which consisted of the same four trading sessions, in the same order, followed by the alternate versions (form B) of the three TASIT-R subscales: EET, SI-M, and SI-E.

Participants signed two separate consent forms for the test and retest treatments (sittings). Taking into consideration the time it took the participants to familiarize with the instructions of the trading task, test and retest sittings each lasted for up to 75 minutes. This does not include the time for completion of the three subscales of the TASIT-R test. On average, the test (retest) sitting, including the three subscales of the TASIT-R test took 120 minutes to complete.

2. Detailed Results

2.1. Descriptive Statistics.

The descriptive statistics for the level of the DE and the scores on TASIT-R subscales (forms A and B) are reported in Table 1.

Table 1. Descriptive statistics
($N = 68$)

Session	DE Mean	DE Std. Deviation	TASIT-R subscales	Mean	Std. Deviation
**S_1**	0.12	0.27	EET(A)	23.04	3.23
**So_1**	0.12	0.28	SI-M(A)	46.12	7.19
**S_2**	0.11	0.28	SI-E(A)	47.19	8.08
**S_3**	0.16	0.25	EET(B)	22.46	2.46
**So_2**	0.12	0.30	SI-M(B)	46.94	6.47
**S_4**	0.08	0.30	SI-E(B)	47.66	6.96

Note: See text for meaning of Session and TASIT-R subscale identifiers

Here, sessions are denoted as follows:

- S_1 is the first trading session, in which participants traded only for themselves;
- O_1 is the second trading session, in which participants traded only for the advisee, who always realized gains (or losses) immediately after the end of the trial (as such, no DE is recorded during this session);
- So_1 is the third trading session, in which participants traded both for themselves and for the advisee;
- S_2 is the fourth trading session, which is identical to S_1, where participants traded only for themselves;
- S_3 is the fifth trading session, and the first trading session of the retest treatment of the study; this session was undertaken four weeks after the completion of the test treatment of the study and is identical to the session S_1;

- O_2 is the sixth trading session, and the second trading session of the retest treatment (identical to O_1);
- So_2 is the seventh trading session, and the third trading session of the retest treatment (identical to So1); and
- S_4 is the eighth trading session, and the fourth and last trading session of the retest treatment (identical to S_2).

Ordinary Least Square (OLS) regression analysis was used to test the joint hypothesis of this study, namely, that transfer of abstraction to non-social domain is possible, and that ToM function builds in part on abstraction, especially when it concerned social-cognitive component of ToM.

To justify the use of OLS, a number of diagnostic checks were performed: histogram and probability plots of standardized residuals, as well as scatterplots of standardized residuals against standardized predicted values, were used to ascertain that the assumptions of normality, linearity, and homoscedasticity of residuals were not violated.

2.2. Predicting the level of DE across trading sessions

OLS regression analysis was used to discover association between individual scores on one of the three TASIT-R subscales, i.e. EET, SI-M, or SI-E, and the level of DE in a session or, alternatively, the change in the level of DE across two sessions. A different version (form A or B) of the TASIT-R subscales was used depending on whether the session pertained to the test or retest treatment. In the analyses below, form A TASIT-R subscales (pertaining to the test treatment) were coded as EET(A), SI-M(A), and SI-E(A); form B subscales (for the retest treatment) were coded as EET(B), SI-M(B), and SI-E(B).

Results of the OLS regression analyses for the level of the DE, per session, including non-standardized (*B*) and standardized (β) regression coefficients for all predictors (the scores on EET(A), EET(B), SI-M(A), SI-M(B), SI-E(A) and SI-E(B) tests), as well as coefficients of determination (R^2),

are reported in Table 2. Here we summarize the results only for those tests which attained significance at the 10% level.

Table 2. Predictors of DE level across trading sessions

Regression coefficients (unstandardized and standardized), standard errors, t-statistics, p-values and coefficients of determination for OLS analyses using *EET*, *SI-M*, and *SI-E* to predict the DE across sessions ($N = 68$).

Session	Predictor variables	***B***	***SE***	***β***	***t***	***p***	R^2	***Lower CI***	***Upper CI***
S_1									
	EET(A)	**0.02**	**0.01**	**0.22**	**1.83**	**0.07**	**0.05**	**-0.002**	**0.04**
	EET(B)	0.001	0.01	0.01	0.09	0.93	0.000	-0.03	0.03
	SI-M(A)	0.01	0.01	0.14	1.17	0.25	0.02	-0.004	0.02
	SI-M(B)	0.01	0.01	0.19	1.58	0.12	0.04	-0.002	0.02
	SI-E(A)	0.01	0.004	0.14	1.11	0.27	0.02	-0.004	0.01
	SI-E(B)	**0.01**	**0.01**	**0.25**	**2.08**	**0.04**	**0.06**	**0.0003**	**0.02**
So_1									
	EET(A)	-0.01	0.01	-0.08	-0.68	0.50	0.01	-0.03	0.01
	EET(B)	0.000	0.01	-0.002	-0.01	0.99	0.000	-0.03	0.03
	SI-M(A)	-0.002	0.01	-0.05	-0.37	0.71	0.002	-0.01	0.01
	SI-M(B)	-0.000	0.01	-0.001	-0.01	0.99	0.000	-0.01	0.01
	SI-E(A)	-0.001	0.004	-0.03	-0.27	0.79	0.001	-0.01	0.01
	SI-E(B)	0.000	0.01	-0.003	-0.03	0.98	0.000	-0.01	0.01
S_2									
	EET(A)	0.01	0.01	0.07	0.60	0.55	0.01	-0.02	0.03
	EET(B)	0.01	0.01	0.11	0.86	0.40	0.01	-0.02	0.04
	SI-M(A)	0.001	0.01	0.02	0.13	0.90	0.000	-0.01	0.01
	SI-M(B)	-0.003	0.01	-0.06	-0.47	0.64	0.003	-0.01	0.01
	SI-E(A)	-0.002	0.004	-0.05	-0.42	0.68	0.003	-0.01	0.01
	SI-E(B)	-0.001	0.01	-0.02	-0.14	0.89	0.000	-0.01	0.01
S_3									
	EET(A)	0.01	0.01	0.13	1.03	0.31	0.02	-0.01	0.03
	EET(B)	0.002	0.01	0.02	0.19	0.85	0.001	-0.02	0.03
	SI-M(A)	**0.01**	**0.004**	**0.28**	**2.33**	**0.02**	**0.08**	**0.001**	**0.02**
	SI-M(B)	**0.01**	**0.004**	**0.29**	**2.48**	**0.02**	**0.09**	**0.002**	**0.02**
	SI-E(A)	**0.01**	**0.004**	**0.30**	**2.52**	**0.01**	**0.09**	**0.002**	**0.02**
	SI-E(B)	**0.01**	**0.004**	**0.28**	**2.37**	**0.02**	**0.08**	**0.002**	**0.02**
So_2									
	EET(A)	0.000	0.01	0.003	0.02	0.98	0.000	-0.02	0.02
	EET(B)	-0.002	0.02	-0.02	-0.15	0.88	0.000	-0.03	0.03
	SI-M(A)	0.01	0.01	0.20	1.66	0.10	0.04	-0.002	0.02
	SI-M(B)	0.01	0.01	0.13	1.08	0.29	0.02	-0.01	0.02
	SI-E(A)	0.01	0.004	0.17	1.42	0.16	0.03	-0.003	0.02
	SI-E(B)	0.01	0.01	0.13	1.05	0.30	0.02	-0.01	0.02
S_4									
	EET(A)	0.01	0.01	0.05	0.44	0.66	0.003	-0.02	0.03
	EET(B)	0.01	0.02	0.04	0.31	0.76	0.001	-0.03	0.03
	SI-M(A)	-0.01	0.01	-0.16	-1.35	0.18	0.03	-0.02	0.003
	SI-M(B)	**-0.01**	**0.01**	**-0.23**	**-1.91**	**0.06**	**0.05**	**-0.02**	**0.0005**
	SI-E(A)	**-0.01**	**0.004**	**-0.23**	**-1.94**	**0.06**	**0.05**	**-0.02**	**0.0002**
	SI-E(B)	**-0.01**	**0.01**	**-0.25**	**-2.08**	**0.04**	**0.06**	**-0.02**	**-0.0004**

In the first trading session ($S_{1,}$), when participants made investment decisions for themselves, EET(A) accounts for a significant 5% variance in the DE; $F(1{,}66) = 3.35, p = 0.07, f^2 = 0.003$ (small effect). Participants' DE increases by 0.02 (see non-standardized B coefficient in Table 2) for each point of the total EET(A) score. Similarly, SI-E(B) is found to be significantly and positively associated with the observed level of DE, accounting for 6% of its variance; $F(1{,}66) = 4.32, p = 0.04, f^2 = 0.004$ (small effect).

In the fifth trading session ($S_{3,}$, the first trading session of the retest sitting), when participants made investment decisions for themselves, SI-M(A) accounts for a significant 8% variance in the DE; $F(1{,}66) = 5.45, p = 0.02, f^2 = 0.006$ (small effect). Likewise, SI M(B) accounts for 9% variance ($F(1{,}66) = 6.17, p = 0.02, f^2 = 0.008$ (small effect)); SI-E(A) for 9% variance ($F(1{,}66) = 6.33, p = 0.01, f^2 = 0.008$ (small effect)); SI-E(B) for 8% variance ($F(1{,}66) = 5.61, p = 0.02, f^2 = 0.006$ (small effect)).

In the eighth trading session (S_4, the fourth and last trading session of the retest sitting), three TASIT-R subscales, i.e. SI-M(B), SI-E(A) and SI=E(B), are found to be significantly and negatively associated with the level of DE observed in S_4: SI-M(B) explains 5% variance of the DE ($F(1{,}66) = 3.65, p = 0.06, f^2 = 0.003$ (small effect)); SI-E(A) for 5% ($F(1{,}66) = 3.77, p = 0.06, f^2 = 0.003$ (small effect)); SI-E(B) for 6% variance ($F(1{,}66) = 4.32, p = 0.04, f^2 = 0.004$ (small effect)).

2.3. Predicting the Change in DE across Trading Sessions

We next investigated the relationship between the scores of three subscales of the TASIT-R tests and the **changes** in the DE between trading sessions. The latter were calculated as post-pre values, which implies that a *negative value reflects a reduction in the DE*. Non-standardized (B) and standardized (β) regression coefficients and coefficients of determination (R^2) are reported in Table 3 and visualized schematically in Figure 6. Here too, we indicate only the significant (at 10% level) findings.

Table 3. Reduction in DE across sessions

Explanatory variables are calculated as post-pre DE values across two trading sessions. Reported are regression coefficients (unstandardized and standardized), standard errors, t-statistics, p-values and coefficients of determination for the OLS analyses using *EET*, *SI-M*, and *SI-E* (tests and retests) as predictors *(N = 68)*.

Post-Pre Difference in DE	Predictor variables	*B*	*SE*	*ß*	*t*	*p*	R^2	*Lower CI*	*Upper CI*
So_1-S_1									
	EET(A)	**-0.03**	**0.01**	**-0.28**	**-2.33**	**0.02**	**0.08**	**-0.05**	**-0.004**
	EET(B)	-0.001	0.02	-0.01	-0.10	0.92	0.000	-0.03	0.03
	SI-M(A)	-0.01	0.01	-0.17	-1.41	0.16	0.03	-0.02	0.003
	SI-M(B)	-0.01	0.01	-0.17	-1.43	0.16	0.03	-0.02	0.003
	SI-E(A)	-0.01	0.01	-0.15	-1.26	0.21	0.02	-0.02	0.003
	SI-E(B)	**-0.01**	**0.01**	**-0.23**	**-1.90**	**0.06**	**0.05**	**-0.02**	**0.001**
S_2- So_1									
	EET(A)	0.01	0.01	0.14	1.14	0.26	0.02	-0.01	0.04
	EET(B)	0.01	0.02	0.10	0.78	0.44	0.01	-0.02	0.04
	SI-M(A)	0.002	0.01	0.05	0.44	0.66	0.003	-0.01	0.01
	SI-M(B)	-0.002	0.01	-0.05	-0.41	0.68	0.003	-0.01	0.01
	SI-E(A)	-0.001	0.01	-0.02	-0.13	0.90	0.000	-0.01	0.01
	SI-E(B)	-0.001	0.01	-0.01	-0.11	0.92	0.000	-0.01	0.01
S_2-S_1									
	EET(A)	-0.01	0.01	-0.12	-1.01	0.32	0.02	-0.04	0.01
	EET(B)	0.01	0.02	0.08	0.68	0.50	0.01	-0.02	0.04
	SI-M(A)	-0.01	0.01	-0.11	-0.89	0.38	0.01	-0.02	0.01
	SI-M(B)	**-0.01**	**0.01**	**-0.22**	**-1.80**	**0.08**	**0.05**	**-0.02**	**0.001**
	SI-E(A)	-0.01	0.01	-0.16	-1.34	0.19	0.03	-0.02	0.003
	SI-E(B)	**-0.01**	**0.01**	**-0.23**	**-1.91**	**0.06**	**0.05**	**-0.02**	**0.0005**
S_3-S_1									
	EET(A)	-0.01	0.01	-0.11	-0.90	0.37	0.01	-0.03	0.01
	EET(B)	0.001	0.01	0.01	0.08	0.94	0.000	-0.03	0.03
	SI-M(A)	0.004	0.004	0.11	0.91	0.37	0.01	-0.01	0.01
	SI-M(B)	0.003	0.01	0.08	0.62	0.54	0.01	-0.01	0.01
	SI-E(A)	0.004	0.004	0.14	1.13	0.26	0.02	-0.003	0.01
	SI-E(B)	0.000	0.01	0.01	0.05	0.96	0.000	-0.01	0.01
S_3-S_2									
	EET(A)	0.003	0.01	0.03	0.26	0.79	0.001	-0.02	0.03
	EET(B)	-0.01	0.02	-0.08	-0.63	0.53	0.01	-0.04	0.02
	SI-M(A)	**0.01**	**0.01**	**0.20**	**1.69**	**0.10**	**0.04**	**-0.002**	**0.02**
	SI-M(B)	**0.01**	**0.01**	**0.28**	**2.39**	**0.02**	**0.08**	**0.002**	**0.03**
	SI-E(A)	**0.01**	**0.01**	**0.28**	**2.37**	**0.02**	**0.08**	**0.002**	**0.02**
	SI-E(B)	**0.01**	**0.01**	**0.24**	**1.98**	**0.052**	**0.06**	**-0.0001**	**0.02**
So_2-S_3									
	EET(A)	-0.01	0.01	-0.09	-0.75	0.45	0.01	-0.03	0.02
	EET(B)	-0.01	0.02	-0.04	-0.28	0.78	0.001	-0.04	0.03
	SI-M(A)	-0.01	0.01	-0.03	-0.21	0.84	0.001	-0.01	0.01
	SI-M(B)	-0.01	0.01	-0.10	-0.83	0.41	0.01	-0.02	0.01
	SI-E(A)	-0.003	0.01	-0.07	-0.54	0.59	0.004	-0.01	0.01
	SI-E(B)	-0.004	0.01	-0.09	-0.77	0.45	0.01	-0.02	0.01
S_4- So_2									
	EET(A)	0.01	0.01	0.05	0.38	0.71	0.002	-0.02	0.03
	EET(B)	0.01	0.02	0.05	0.42	0.68	0.003	-0.03	0.04
	SI-M(A)	**-0.02**	**0.01**	**-0.33**	**-2.81**	**0.01**	**0.11**	**-0.03**	**-0.004**
	SI-M(B)	**-0.02**	**0.01**	**-0.32**	**-2.76**	**0.01**	**0.10**	**-0.03**	**-0.01**
	SI-E(A)	**-0.02**	**0.01**	**-3.62**	**-3.16**	**0.002**	**0.13**	**-0.02**	**-0.01**
	SI-E(B)	**-0.02**	**0.01**	**-0.34**	**-2.90**	**0.01**	**0.11**	**-0.03**	**-0.01**
S_4-S_3									
	EET(A)	-0.01	0.01	-0.04	-0.36	0.72	0.002	-0.03	0.02
	EET(B)	0.002	0.02	0.02	0.14	0.89	0.000	-0.03	0.04
	SI-M(A)	**-0.02**	**0.01**	**-0.34**	**-2.97**	**0.004**	**0.12**	**-0.03**	**-0.01**
	SI-M(B)	**-0.02**	**0.01**	**-0.41**	**-3.66**	**0.001**	**0.17**	**-0.03**	**-0.01**
	SI-E(A)	**-0.02**	**0.01**	**-0.42**	**-3.72**	**0.001**	**0.17**	**-0.03**	**-0.01**
	SI-E(B)	**-0.02**	**0.01**	**-0.42**	**-3.74**	**0.001**	**0.18**	**-0.03**	**-0.01**
S_4-S_1									
	EET(A)	-0.01	0.01	-0.12	-1.00	0.32	0.02	-0.04	0.01
	EET(B)	0.003	0.02	0.02	0.18	0.86	0.001	-0.03	0.04
	SI-M(A)	**-0.01**	**0.01**	**-0.24**	**-2.03**	**0.046**	**0.06**	**-0.02**	**-0.0002**
	SI-M(B)	**-0.02**	**0.01**	**-0.33**	**-2.86**	**0.006**	**0.11**	**-0.03**	**-0.01**
	SI-E(A)	**-0.01**	**0.01**	**-0.29**	**-2.49**	**0.015**	**0.09**	**-0.02**	**-0.003**
	SI-E(B)	**-0.02**	**0.01**	**-0.39**	**-3.46**	**0.001**	**0.15**	**-0.03**	**-0.01**

When testing the reduction in the DE *between the first trading session* (trading for oneself, S_1) *and the third trading session* (trading both for oneself and others, So_1), i.e. $(So_1\text{-}S_1)$, EET(A) and SI-E(B) are found to account for a significant *reduction* of the DE explaining 8% and 5% of the variation in the change ($F(1,66) = 5.43, p = 0.02, f^2 = 0.006$ (small effect); and $F(1,66) = 3.59, p = 0.06, f^2 = 0.003$ (small effect), respectively).

Next, SI-M(B) and SI-E(B) are found to explain the *reduction* in the DE *between the first and the fourth trading sessions*, i.e. $(S_2\text{-}S_1)$, on marginally significant levels with 5% and 5% of variance explained ($F(1,66) = 3.22, p = 0.08, f^2 = 0.003$ (small effect); and $F(1,66) = 3.65, p = 0.06), f^2 = 0.003$ (small effect).

The analysis of the change in DE before and after the four-week test-retest interval shows a pronounced treatment washout. Thus, between *the fourth trading session and the fifth trading session* (i.e. $(S_3\text{-}S_2)$), SI-M(A), SI-M(B), SI-E(A) and SI-E(B) are found to be significantly associated with an *increase* in the DE. SI-M(A) explains 4% of the variance in the increase of the DE ($F(1,66) = 2.85, p = 0.096, f^2 = 0.002$ (small effect)). SI-M(B) explains 8% of the variation in the change ($F(1,66) = 5.73, p = 0.02, f^2 = 0.006$ (small effect)); SI-E(A) explains 8% of the variance ($F(1,66) = 5.59, p = 0.02, f^2 = 0.006$ (small effect)); SI-E(B) explains 6% of the variance ($F(1,66) = 3.91, p = 0.052, f^2 = 0.004$ (small effect)).

Between *the seventh trading session* and *the eighth trading session* (i.e. $(S_4\text{- }So_2)$), SI-M(A), SI-M(B), SI-E(A), and SI-E(B) all account for a significant *reduction* in the DE, with 11%, 10%, 13% and 11% of the variance explained, respectively. The F-statistics and significance level(s) are $F(1,66) = 7.87, p = 0.01, f^2 = 0.012$ (small effect) for SI-M(A); $F(1,66) = 7.63, p = 0.01, f^2 = 0.01$ (small effect) for SI-M(B); $F(1,66) = 9.98, p = 0.002, f^2 = 0.017$ (small effect) for SI-E(A); $F(1,66) = 8.43$, $p = 0.01, f^2 = 0.012$ (small effect) for SI-E(B).

Between *the fifth trading session* and *the eighth trading session*, both in the retest treatment ((i.e. $(S_4\text{- }S_3)$), SI-M(A), SI-M(B), SI-E(A), and SI-E(B) again are found to explain a significant

fraction of the reduction in the DE, with 12%, 17%, 17%, and 18% of the variance explained. The F-statistics and significance level(s) for SI-M(A) are $F(1,66) = 8.79, p = 0.004, f^2 = 0.015$ (small effect); for SI-M(B) are $F(1,66) = 13.42, p < 0.001, f^2 = 0.03$ (small effect); for SI-E(A) are $F(1,66) = 13.86$, $p < 0.001, f^2 = 0.03$ (small effect); and for SI-E(B) are $F(1,66) = 14.01, p < 0.001, f^2 = 0.033$ (small effect).

When predicting the improvement in the DE between *the first trading session* (playing for oneself) and *the eighth trading session* (the last trading session, playing for oneself) (i.e. $(S_4 - S_1)$), SI-M(A) accounted for a significant 6 % variance in the change of DE, $F(1,66) = 4.13, p = 0.046, f^2 = 0.004$ (small effect). SI-M(B) accounted for a significant 11 % variance in the change of DE, $F(1,66) = 8.21, p = 0.006, f^2 = 0.012$ (small effect). SI-E(A) accounted for a significant 9% variance in the change of DE, $F(1,66) = 6.22, p = 0.015, f^2 = 0.008$ (small effect). SI-E(B) accounted for a significant 15% variance in the change of DE, $F(1,66) = 11.93, p < 0.001, f^2 = 0.023$ (small effect).

2.4 Testing for the reduction in DE across high- and low-ToM Groups

The results reported above demonstrate that individuals with higher social-cognitive component of ToM measured using SI-M and SI-E subscales of the TASIT-R test were more responsive to the ToM-based intervention, while higher scores on the EET test, which operationalizes the level of social-perceptual ToM, did not lead to a significant cognitive training effect. As shown in Table 2 and Figure 5, among the two TASIT-R social inference subscales, only SI-E consistently (which means: based on both the form A and form B scores) predicted the reduction in the DE upon the second intervention.

To provide a more nuanced (less dependent on linearity than regression) understanding of the changes in the DE across low- and high-ToM groups, we averaged the test-retest scores across the form A and B SI-E tests, after which we conducted a median split[21]. The mean levels of DE before and after interventions in the low- and high-TOM groups are shown in Figure 5.

A series of paired-samples *t*-tests were conducted to test for changes in DE scores pre- and post-intervention in the resulting low- and high-ToM groups. We verified that the assumption of homogeneity of variance was met for DE scores across all sessions depicted in Figure 5, and for both groups.

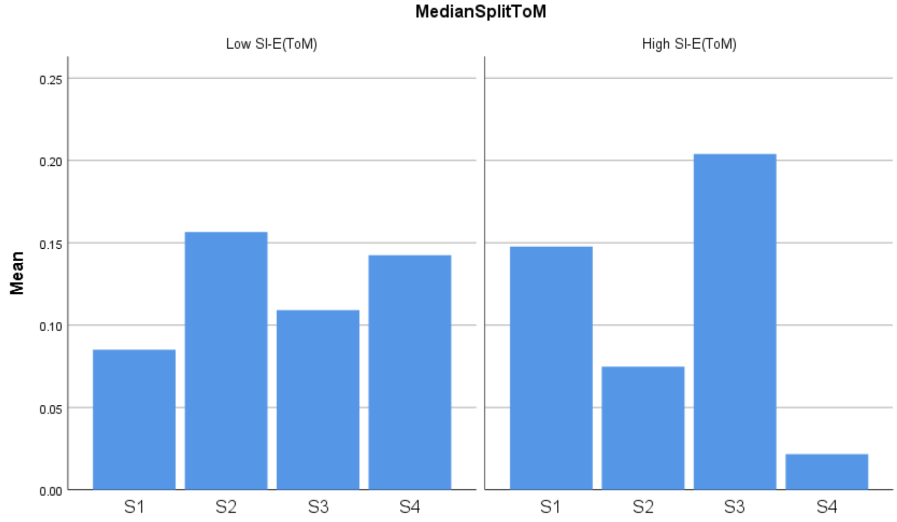

Figure 5. Mean DE levels in 'trading for oneself' sessions stratified by ToM scores
ToM groups are formed based on the median split of the mean scores of SI-E subscale (forms A and B) of the TASIT-R test.

No significant differences are observed in left-tailed, paired-samples *t*-tests (whether average *post pre* DE score reduction is equal to zero) in the *low social-cognitive component of ToM* group ($p >$ 0.10 for the following tests: S_3 and S_2, $t(32) = -1.12$; S_4 and S_3, $t(32) = 0.82$; S_4 and S_1 $t(32) = 1.26$). A significant increase in disposition effect is observed in *low-ToM* group after the first intervention (S_2 and S_1, $t(32) = 1.78$; $p = 0.043$, left-tailed, $d = 0.30$ (small effect)), thus rejecting the null of no effect.

No significant effect emerges for the *high-ToM* group between sessions S_2 and S_1 ($t(34) = -1.16$, $p = 0.13$). Between sessions S_3 and S_2 in the high-ToM group, the difference is significant and positive: $t(34) = 2.19$, $p = 0.018$, left-tailed, $d = 0.45$ (small effect), thus rejecting the null of no effect. A *lower* mean disposition score is observed in session S_2 ($M = 0.07$, $SD = 0.30$) which followed the first

intervention, than in session S_3 (M = 0.20, SD = 0.27), the first session after a four-week treatment washout period. A significant *reduction* in DE in the *high-ToM* group is recorded after the second intervention, i.e. between sessions S_3 and S_4 ($t(34)$ = -2.75, p = 0.005, left-tailed, $d = -0.59$ (medium effect)) in the high-ToM group. The mean disposition score is lower in session S_4, the last session of the experiment (M = 0.02, SD = 0.35), than in session S_3, the last session of the test treatment (M = 0.20, SD = 0.27). The difference of 0.18 amounts to an 89.4% reduction in the DE.

Finally, the comparison of scores obtained in the first (S_1) and last (S_4) trading sessions of the experiment reveals an *85.4% reduction* in the DE; it is significant ($t(34)$ = -1.79, p = 0.042, left-tailed, $d = -0.38$ (small effect)).

2.5. Isolating the Mechanism Leading to Reduction in DE Among Participants with an Above-Median Score of Social-Cognitive Component of ToM

Next, we investigated whether and when successful trainees stop paying attention to the cue that is key to the DE, namely, the purchase price. We measured the selective aspects of participants' attention using eye gaze[22-23]: if a participant's eyes were oriented towards an object (e.g., the purchase price), we assumed that she is paying more attention to the object than to other elements of the screen. Following Holmqvist and collaborators[24], total fixation count on the trading dashboard was used as the base measure of eye gaze fixations, and fixations on an area of interest (AOI) was counted against this base. We also investigated total fixation durations (total dwell time). This was found to yield the same results (including significance levels) to the ones based on the ratio of fixation counts reported here. Therefore, one AOI is the cell in the trading dashboard displaying the acquisition price (AOI_AP); the other ones are: AOI_DB (entire trading Dashboard), AOI_APS (Own Acquisition Price), AOI_APO (Advisee's Acquisition Price). Our measures captured the degree of attention to the AOI, normalized for total attention paid to all information presented on the trading dashboard. Herein, the reduction of individual attention to the information presented on the trading dashboard may either be associated with individual's disengagement with the task or individual's thought

processing which did not necessitate the regular attendance of the information cues presented on the trading dashboard.

In Table 4, descriptive statistics on eye gaze results are presented. A number of independent-samples *t*-tests on the eye gazes will now be discussed. These test for significance of differences in several eye gaze ratios across sessions, as well as differences across (high and low social-cognitive component of ToM) groups.

Table 4. Eye Gazes.

Descriptive statistics associated with the eye tracking measures ($N = 63$).

Eye tracking measure	Mean (Count or Ratio)	Std. Deviation
**S_1_AOI_AP**	272.98	115.13
**S_1_AOI_DB**	2251.75	586.11
**S_1_AOI_ratio_APDB**	0.13	0.05
**So_1_AOI_APO**	85.46	54.96
**So_1_AOI_APS**	200.51	102
**So_1_AOI_DB**	2302.78	617.11
**So_1_AOI_ratio_APODB**	0.04	0.03
**So_1_AOI_ratio_APSDB**	0.09	0.04
**S_2_AOI_AP**	234.25	111.88
**S_2_AOI_DB**	1978.60	510.35
**S_2_AOI_ratio_APDB**	0.12	0.05
**S_3_AOI_AP**	227.60	119.01
**S_3_AOI_DB**	2125.90	586.34
**S_3_AOI_ratio_APDB**	0.11	0.05
**So_2_AOI_APO**	65.90	47.57
**So_2_AOI_APS**	153.86	88.35
**So_2_AOI_DB**	2216.11	586.79
**So_2_AOI_APODB**	0.03	0.02
**So_2_AOI_APSDB**	0.07	0.04
**S_4_AOI_AP**	184.51	125.46
**S_4_AOI_DB**	1943.29	523.14
**S_4_AOI_ratio_APDB**	0.10	0.06

Notes:
(a) The titles for eye tracking measures consist of (i) *Session* (S_1, So_1,…, S_4), (ii) *Area of Interest* 'AOI', (iii) *level* (e.g. Acquisition Price 'AP', Own Acquisition Price 'APS' (i.e. 'Acquisition Price – Self"), Advisee's Acquisition Price 'APO' (i.e. 'Acquisition Price – Other'), or entire Dashboard 'DB'), or *ratio* (e.g., ratio_APDB stands for 'ratio of AP over DB').
(b) For So1_AOI_APO: fixation count on the AOI associated with the acquisition price of the security when trading for others (in the condition when the participants can trade for themselves and others).
(c) For So1_AOI_APS: Fixation count on the AOI associated with the acquisition price of the security when trading for oneself (in the condition when the participants can trade for themselves and others).

In session S_1, many differences between the groups representing high and low social-cognitive component of ToM are insignificant, such as the ratio of the count of fixations on the acquisition price and on the dashboard (S_1_AOI_ratio_APDB). The lack of significant group differences is also observed in the session where participants trade both for themselves and others (So_1), for (i) the ratio between count of fixation on (own) acquisition price and on dashboard (So_1_AOI_ratio_APSDB), and (ii) the ratio between count of fixation on the advisee's acquisition price and on dashboard (So_1_AOI_ratio_APODB).

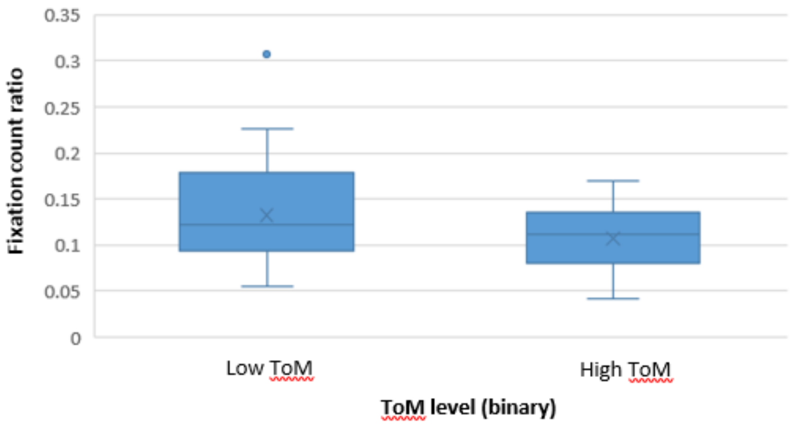

Figure 6. Differences in attention to own acquisition price across the levels of social-cognitive component of ToM in the last session of the test treatment (S_2)
Shown are boxplots of the ratios of count of fixations on the acquisition price to the count of fixations on the entire trading dashboard (S_2_ratio_APDB), separately for participants with high- and low social-cognitive component of ToM, Session S_2.

In the last session of the test treatment (S_2), a significant group difference is observed for the ratio of the count of fixations on the acquisition price to the count of fixations on the dashboard (S_2_AOI_APDB), $t(61) = 2.18$, $p = 0.033$, two-tailed. Figure 6 depicts this in a boxplot.

In the first experimental session of the retest treatment (coded as Session S_3), no significant group differences are recorded for the ratio between count of fixation on the acquisition price and on the dashboard (S_3_AOI_ratio_APDB). In contrast, in the session of the retest treatment where participants trade both for themselves and others (So_2_AOI_APSDB), highly significant differences

between high and low social-cognitive component of ToM groups are found ($t(61) = 3.83$, $p < 0.001$, two-tailed, $d = 0.99$ (large effect)). This is shown graphically with a boxplot in Figure 7. The same applied to the ratio of fixation count on the advisee's acquisition price against total fixation count (So_2_AOI_APODB) ($t(61) = 3.49$, $p = 0.001$, two-tailed, $d = 0.94$ (large effect)). Figure 8 displays the corresponding boxplots. Finally, in the last trading session of the experiment (S_4_AOI_APDB), highly significant group differences in counts of relative fixation on the acquisition price emerged, $t(61) = 4.94$, $p < 0.001$, two-tailed, $d = 1.34$ (large effect); see Figure 9.

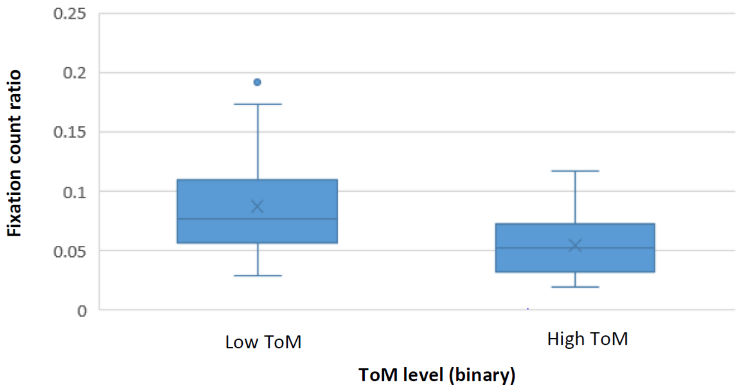

Figure 7. Differences in attention to own acquisition price across social-cognitive component of ToM levels in the third session after washout period (So_2)
Displayed are boxplots of the ratios of count of fixations on own acquisition price to the count of fixations on the trading dashboard, stratified by participants' social-cognitive component of ToM score, Session So_2.

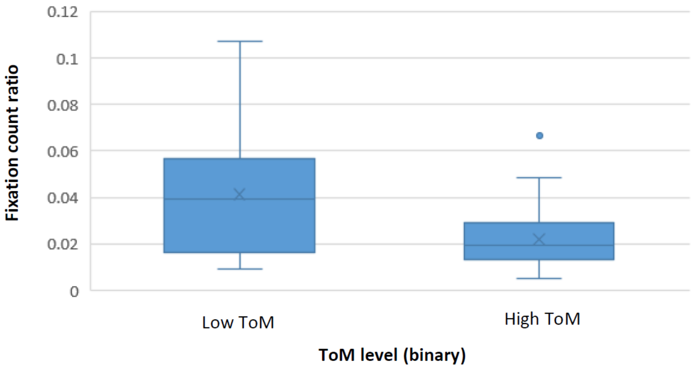

Figure 8. Differences in attention to advisee's acquisition price across social-cognitive component of ToM levels in the third session after washout period (So_2)

Displayed are boxplots of the ratios of count of fixations on advisee acquisition price to the count of fixations on the trading dashboard, stratified by participants' social-cognitive component of ToM, Session So2.

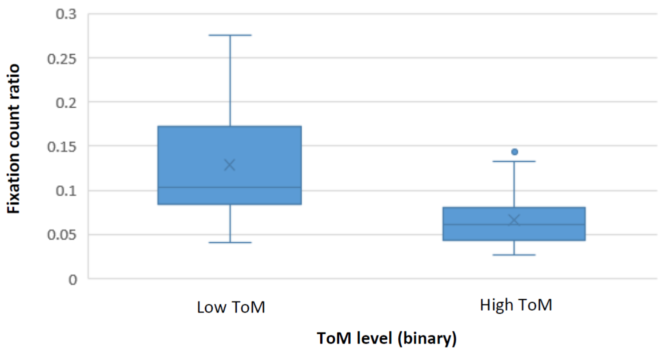

Figure 9. Differences in attention to acquisition price across social-cognitive ToM levels, last session of the experiment (S_4)

Displayed are boxplots of the ratios of count of fixations on the acquisition price to the count of fixations on the trading dashboard, stratified by participants' social-cognitive ToM score, Session S_4.

Next, we report within-group counts of gaze fixation on the acquisition price before and after the interventions in test and retest sittings.

In the *low* social-cognitive component of ToM group, there was no significant difference in the number of fixations on the acquisition price relative to total number of fixations on the trading dashboard between S_2 and S_1, between S_4 and S_3, and between S_4 and S_1..

In the group representing *high* social-cognitive component of ToM, there was a significant decline in the number of fixations on the acquisition price relative to total number of fixations on the trading dashboard after the experimental interventions in both test and retest sittings. After the first intervention (S_2-S_1), the above ratio declined by 11.4% ($t(31) = 2.57$, $p = 0.015$, two-tailed, $d = 0.37$ (small effect)); after the second intervention (S_4-S_3), the ratio declined by 36.6% ($t(31) = 6.03$, $p < 0.001$, two-tailed, $d = 1.08$ (large effect)). Overall, from the first to the last experimental sessions (S_4-S_1), the ratio of count of fixations on the acquisition price to the count of fixations on the trading dashboard decreased by 45.1% ($t(31) = 6.40$, $p < 0.001$, two-tailed, $d = 1.59$ (large effect)).

Altogether, these findings show that participants who demonstrated high level of social-cognitive component of ToM overall paid less attention to the acquisition price already from the first intervention. This effect was magnified after the four-week washout period, despite a temporary increase in the DE in the first session of the retest treatment. Eye gaze evidently followed the same pattern as the DE score, suggesting that reductions in the DE could be attributed to attention that is turned away from irrelevant information, in this case, the acquisition price.

2.6 Post hoc analyses

Finally, we conducted post hoc analyses to investigate the role of the trading experience on the proneness to DE across experimental sessions. Multiple regression analyses with ToM score and trading experience as predictor variables revealed no significant effect of trading experience on change in DE, calculated as post-pre DE. When considering the effect of trading experience on DE level, we found that participants with trading experience had a significantly ($p < 0.05$) higher level of DE in session (IV) of the first sitting and in sessions (I) and (III) of the second sitting, while in other sessions the effect of trading experience on DE was not significant. Controlling for trading experience while testing the effect of social-perceptual and social-cognitive components of ToM on the level of DE and post-pre change in DE generally resulted in more significant findings for the tests reported as significant. However, this did not add any extra insights to what is already reported in the findings.

INTERNET APPENDICES

APPENDIX A: Technical specifications of the eye tracking measurements

Core parameters	Parameter description	Parameter specifications adopted in the experiment
**Measures**	Definition of eye tracking measures	Fixation count, i.e. the number of times the participant fixates on an AOI, is adopted as the base eye tracking measure. In this study, we used the composite eye tracking measure, which is the ratio of the fixation count on the AOI associated with the purchasing price to the AOI denoting the number of fixations on the trading dashboard.
**Apparatus**	Sampling procedure	Binocular recording procedure was used (i.e. pupil dilation and eye tracking measures are based on the data acquired from both left and right eyes of the participants)
	Name and produce of the eye tracking device	Tobii TX300, Tobii (Sweden)
	Type of eye tracking device	Desk-mounted
	Sampling rate	300 Hz
	Sampling rate variability	0.3%
	Processing latency	1.0 – 3.3 ms
	Accuracy (the angular average distance from the actual gaze point to the one measured by the eye tracker)	0.4^0 – at ideal conditions, 0.3^0 - at 25^0 gaze, 0.6^0 - at 30^0 gaze, 0.6^0 – at 1 lux, 0.4^0 – at 300 lux, 0.5^0 – at 600 lux, 0.5^0 – at 1000 lux.
	Precision	0.01^0 – with Stamper filter (for more details on the applied Stampe algorithm for noise reduction see [25])
	Eye tracking software used	Tobii Studio 3.4.5
	Chin rest used	Yes
**Monitor**	Screen size	23'''
	Screen resolution	1920 x 1080 pixel
	Distance between participant and screen	Operating distance: 50-80cm<br>Default distance used in this study: 65cm
**Calibration**	How many points in calibration	9-point calibration
	Amount of recalibration	No recalibration used
**Materials**	Example image included?	Yes (see Figures 1-4 and Appendix B)
	Participant vision (corrected or not)	corrected-to-normal vision.
**Areas of Interest (AOIs)**	AOIs used for eye tracking data analysis	The list of AOIs is reported in Table 4 and illustrated in Figures B1, B2, B3 (Appendix B)
**Exclusions**	Number of trials excluded	None

	Number of participants excluded due to the missing eye tracking data	5 participants (7.35% of the behavioral sample)
	Data quality threshold	A data quality threshold of 85% was used, i.e. at least 85% of the eye tracking data for the practice and for all of the trading sessions had to be present, otherwise the participant was excluded from the sample.
**Event detection**	What algorithm is used for event detection	The IV-T fixation [19] was adopted via the selection of global settings in the eye tracking software (Tobii Studio 3.4.5). While the minimal length of fixations did not play a major role in the calculation of processing speed in our study, a rather conservative 60ms threshold was selected within IV-T Tobii filter parameters to define fixations.

APPENDIX B: Visual representation of the areas of interest used in the eye tracking analysis

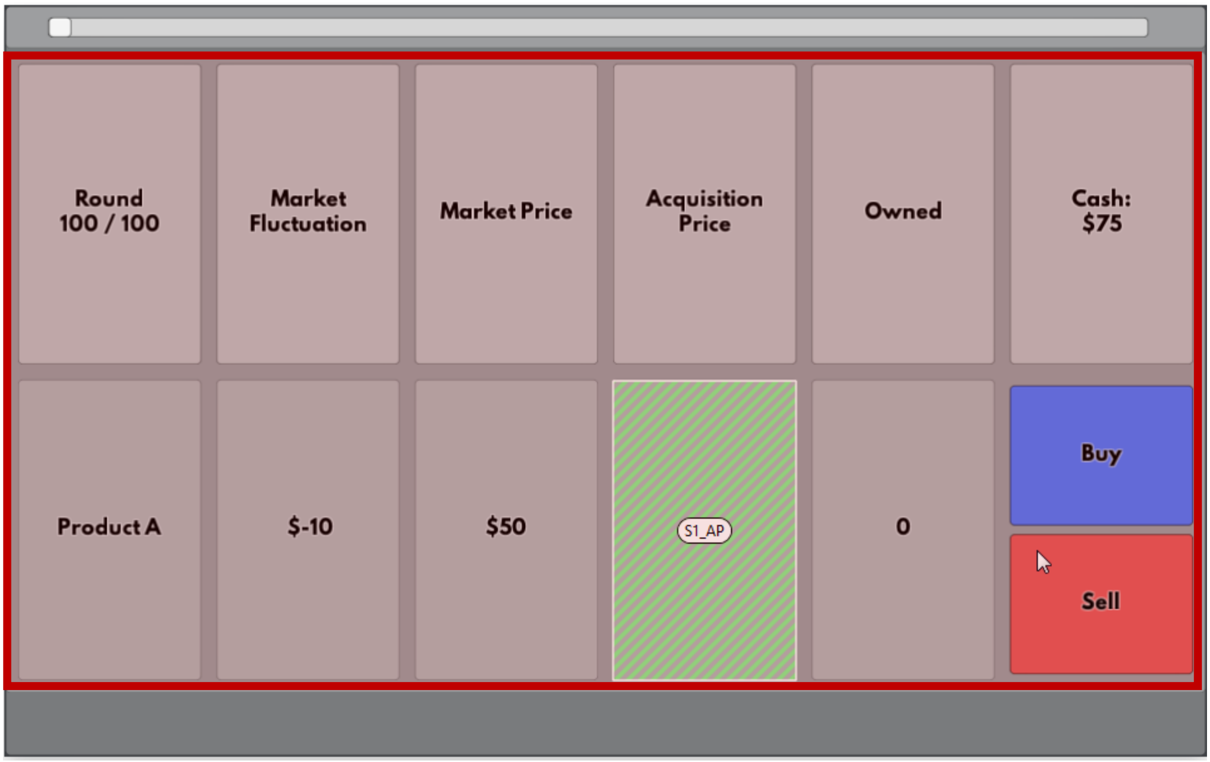

Figure B1. AOIs on the version of the trading dashboard where participants trade for themselves; a smaller AOI covers the area where the acquisition price is indicated, whereas the larger AOI covers the whole dashboard (excluding the progress bar).

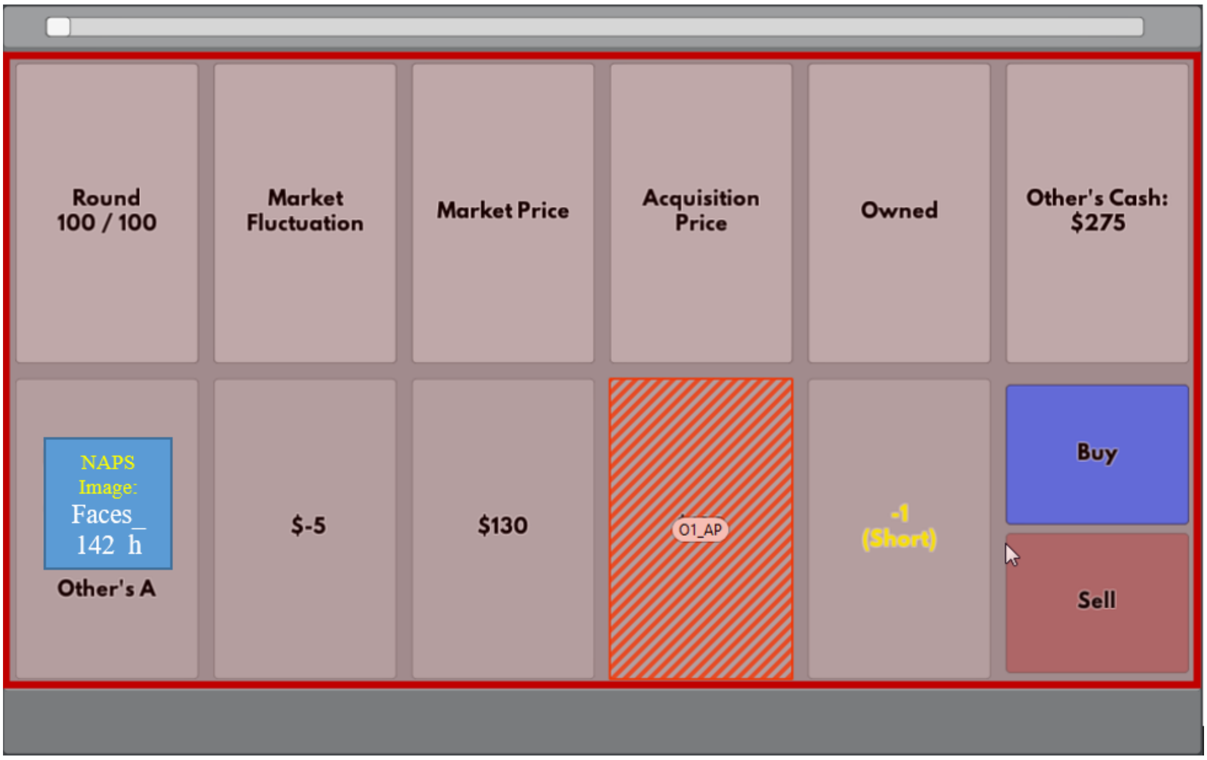

Figure B2. AOIs on the version of the trading dashboard where participants trade for others; a smaller AOI covers the area where the acquisition price is indicated, whereas the larger AOI covers the whole dashboard (excluding the progress bar).

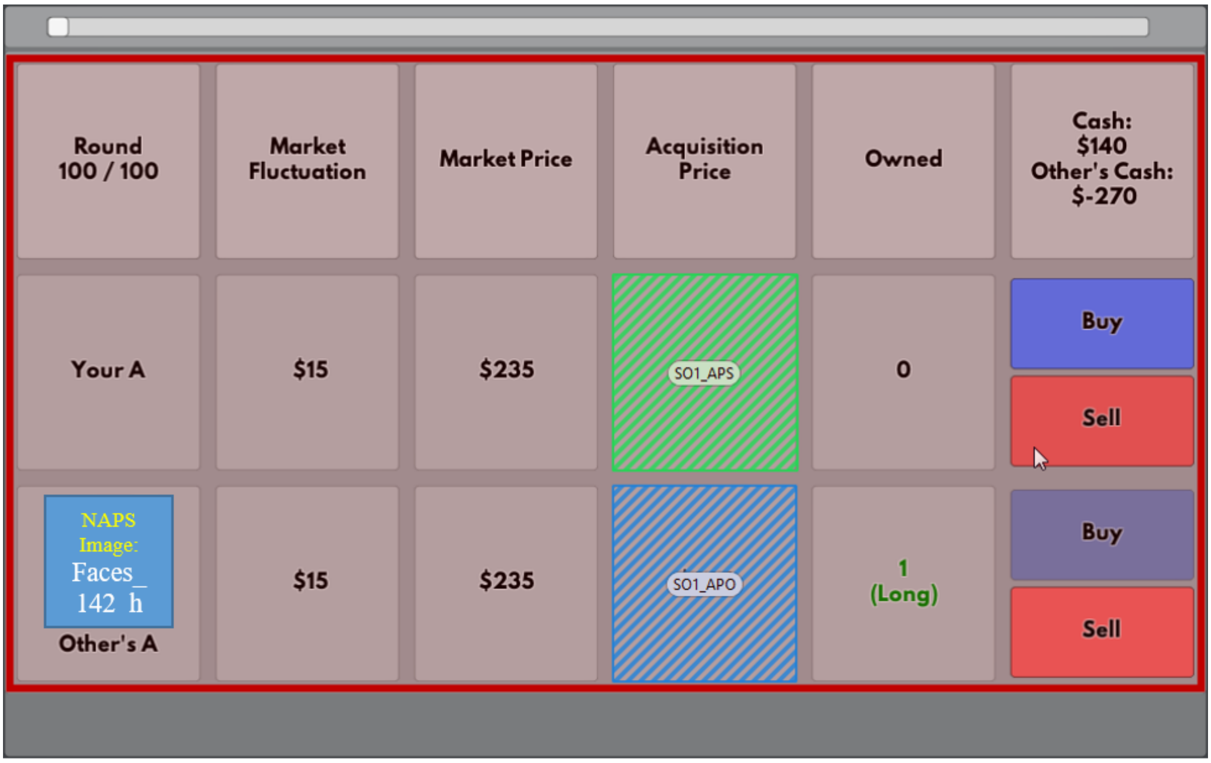

Figure B3. AOIs on the version of the trading dashboard where participants trade for both themselves and others; two smaller AOIs cover the areas where the acquisition price of the security purchased for oneself ('Your A') and others ('Other's A') is indicated, whereas the larger AOI covers the whole dashboard (excluding the progress bar).

Note: Due to copyright restrictions, the original images that were used in the experiment cannot be reproduced here. However, as an example, we report the image code/title, as per Nencki Affective Picture System (NAPS)

APPENDIX C: Instructions supplementing the experimental task (Disposition Game)

(Note: the titles in curly brackets are included for explanatory purposes only and were not seen by the participants)

{Introductory instructions}

Disposition Game

In the Disposition Game, you will attempt to maximise profits while buying and selling a security and avoiding the DE.

Gameplay

Each round consists of the time it takes for the bar at the top of the game screen to run out (approx. 10 secs). Once the time is over, a new round is started. The timing between rounds is minimal (less than 1 second). During each round, a player can buy or sell exactly 1 security. Player's decision (submit an order to buy or sell the security, or keep the current position) is not submitted until the end of the round.

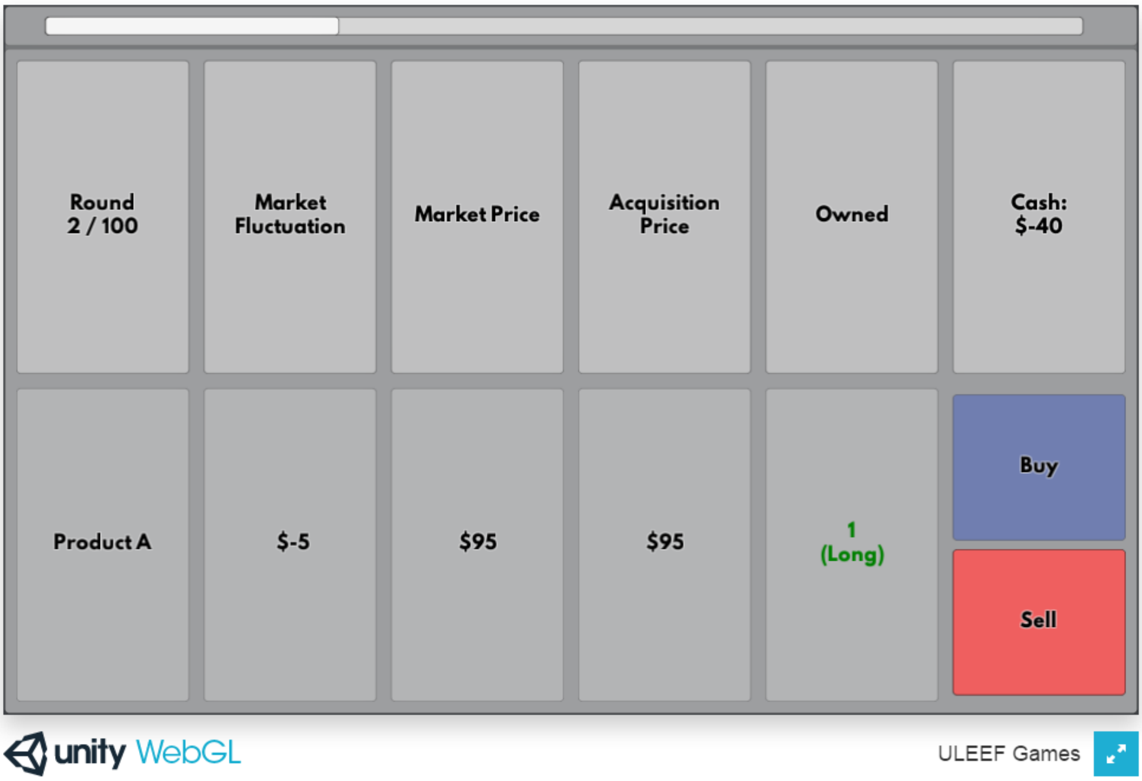

Next to the security (in this case, Product A) you will see a series of values (see above):

- **Market Fluctuation** is how much the market price has changed since the last round.

- **Market Price** is the current value of the stock if it were bought or sold.

- **Acquisition Price** is the price you purchased or short-sold the stock at last.

- **Owned** is the current amount of units of the security owned by the player.

Suppose that you are playing the Disposition Game .Once the round started and you made a decision (for example, to Buy the Product A), you may cancel your trade by pressing the "Sell" button, or you may subsequently short sell the stock by pressing "Sell" button again as it is demonstrated on two screenshots below:

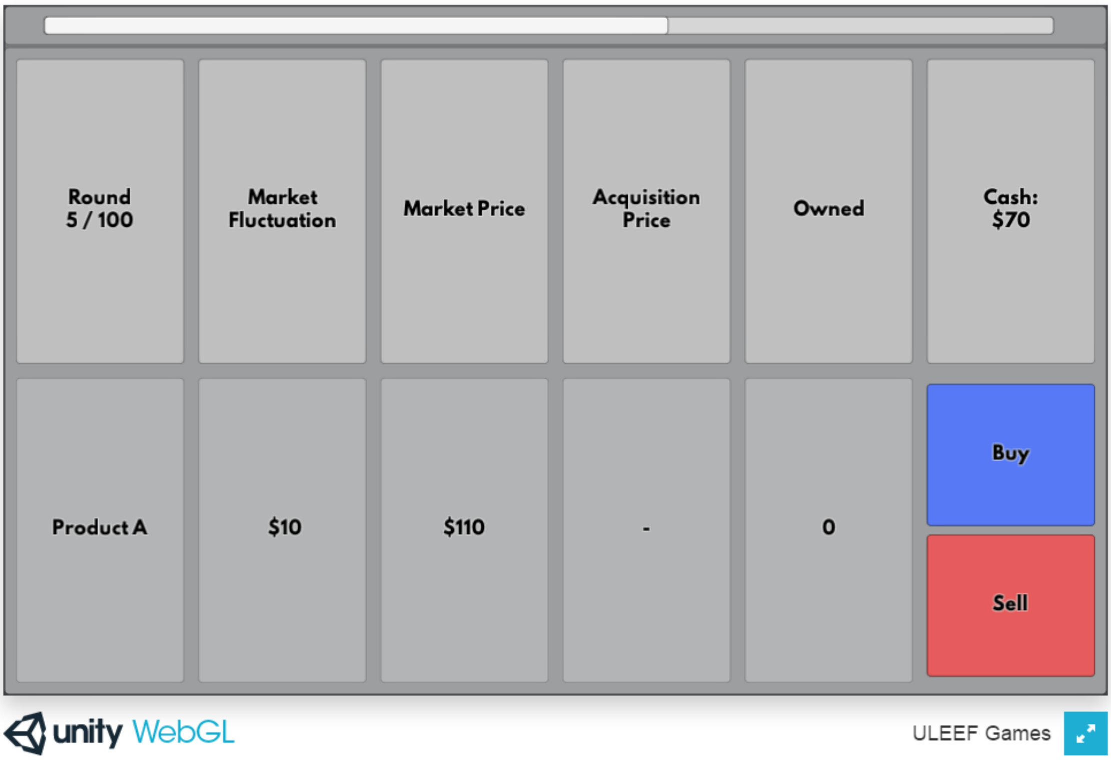

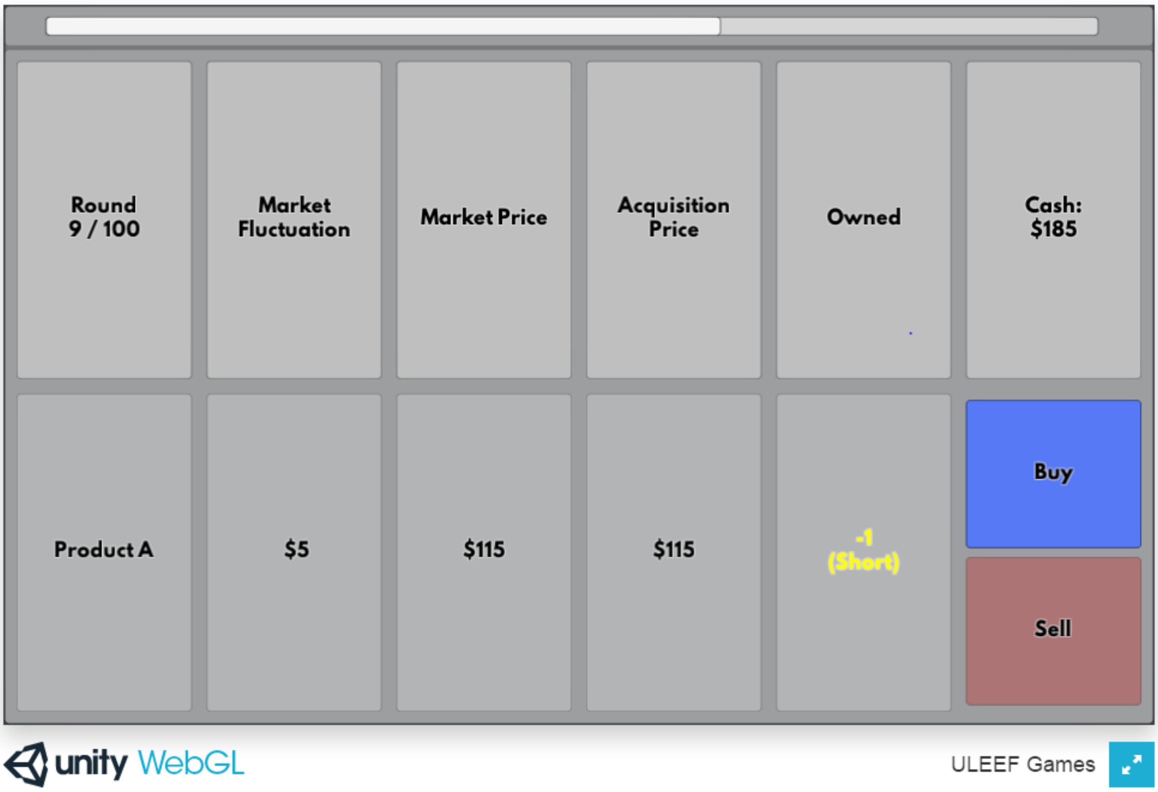

The last trade before the game round finishes is considered to be your actual trade for that round. To sum up, you can undo any decision to buy or sell your security until the end of the round.

The overall game consists of four trading sessions: one practice trading session (30 rounds) to familiarise yourself with the interface of the trading game and four consecutive main trading sessions (100 rounds each), which are going to be described in more details later.

Next, you will proceed to the practice trading session.

{Instructions preceding the practice session}

You are about to begin the practice trading session. The session will consist of 30 rounds. In each round, you will have ten seconds to place an order (Buy or Sell) for one security available in the experimental market (Product A). Before the bar at the top of the game screen runs out, you can change your order. The following information is going to be provided to you on the dashboard:

- **Market Fluctuation** - how much the market price has changed since the last round.

- **Market Price** - the current value of the stock if it were bought or sold.

- **Acquisition Price** - the price you purchased or short-sold the stock at last.

- **Owned** - the current amount of units of the security owned by the player.

At the end of the practice trading session, you will be shown the amount earned for the session and the DE score that characterises your investment behaviour (the higher is the value, the greater is the DE). The dollar amount is provided in the end of the practice round as another indicator of how successful the selected trading strategy was. As it is just a practice trading session, this money is not going to be paid to you. However, at the end of each main trading session, the indicated dollar amount will reflect how much you actually earned throughout the session. This dollar amount will form the basis of the take-home amount that is going to be paid to you in cash at the end of today's experimental session.

For your information, at the end of the main experiment, the take-home amount will be calculated as follows. If the total value of your cash and risky asset holdings at the end of the main trading session 1 is X_1, the total value of your cash and risky asset holdings at the end of the session 2 is X_2, at the end of session 3 is X_3, and at the end of session 4 is X_4 (in experimental currency), then your take-home pay in actual dollars is equal to $10 + (X_1 + X_2 + X_3 + X_4)/48$ (in Australian dollars).

This amount will be paid to you in cash at the end of today's experiment.

Before moving forward, please raise your hand if you have any questions and the research facilitator will assist you.

{Instructions preceding the session S_1 in test treatment and session S_3 in retest treatment}

You are about to begin the first main trading session. This session repeats the design of the practice trading session, however it now consists of 100 trading rounds. Each round is 10 secs long. The amount of dollars earned (or lost) does not roll over to the following trading session(s). Thus, at the end of each trading session, your holdings of the Product A will be liquidated and the cash value of your position will be recorded. If you finish the trading session with the negative amount, you will not be required to pay this amount and your actual balance will be nullified before the next trading session.

{Instructions preceding the session O_1 in test treatment and session O_2 in retest treatment}

You are about to begin the second main trading session. In this session, you will help to make investment decisions to one of the clients of the company you are working for. Before the session begins, you will be provided with the photographs from client profiles. You will be asked to select one photograph of the client you would be helping by providing financial advice, i.e. by placing the orders for the customer. Thus, in this session you will be trading the same security, but now you will be doing it for the client. Based on your trading performance, you will be awarded a fraction (25%) of the client's earnings.

{Instructions preceding the session So_1 in test treatment and session So_2 in retest treatment}

You are about to begin the third trading session. In this session, you will have an option to place trades both for yourself and/or for your client. As in previous trading sessions, in each round you will have an option to place multiple orders before the time is up. The result of trading for yourself as well as for trading for your client (25% of the client's earnings) will be included in your reward for this trading session.

{Instructions preceding the session S_2 in test treatment and session S_4 in retest treatment}

You are about to begin the fourth and last trading session. As in the first main trading session, you are going to make investment decisions for yourself only, while trading one security. Remember, you can make multiple trading decisions within one round.

REFERENCES

1. Frydman, Cary, Nicholas Barberis, Colin Camerer, Peter Bossaerts, and Antonio Rangel, 2014, Testing theories of investor behavior using neural data, *Journal of Finance* 69, 907–946.
2. Odean, Terrance, 1998, Are investors reluctant to realize their losses? *Journal of Finance* 53, 1775–1798.
3. van Rijn, Hedderik, Jelle R. Dalenberg, Jelmer P. Borst, and Simone A. Sprenger, 2012, Pupil dilation co-varies with memory strength of individual traces in a delayed response paired-associate task. *PLoS One*, *7*(12), e51134.
4. Chiew, Kimberly Sarah, and Todd S. Braver, 2013, Temporal dynamics of motivation-cognitive control interactions revealed by high-resolution pupillometry. *Frontiers in Psychology, 4* .1-15.
5. Frydman, Cary, and Antonio Rangel, 2014, Debiasing the disposition effect by reducing the saliency of information about a stock's purchase price, *Journal of Economic Behavior & Organization* 107, 541–552.
6. Harris, Anthony D., Jessina C. McGregor, Eli N. Perencevich, Jon P. Furuno, Jingkun Zhu, Dan E. Peterson, and Joseph Finkelstein, 2006, The use and interpretation of quasi-experimental studies in medical informatics. *Journal of the American Medical Informatics Association* 13 (1), 16-23.
7. Goulart, Marco, Newton C.A. da Costa Jr, Eduardo B. Andrade, and André A.P. Santos, 2015, Hedging against embarrassment. *Journal of Economic Behavior & Organization*, *116*, 310-318.
8. Fischbacher, Urs, Gerson Hoffmann, and Simeon Schudy, 2017, The causal effect of stop-loss and take-gain orders on the disposition effect. *The Review of Financial Studies*, *30*(6), 2110-2129.
9. Flanagan, Sharon, Skye McDonald, Jennifer Rollins, 2011, *The Awareness of Social Inference Test – Revisited (TASIT-R)*. Australia: University of New South Wales, Sydney.
10. Tager-Flusberg, Helen, and Kate Sullivan, 2000, A componential view of theory of mind: evidence from Williams syndrome. *Cognition*, *76*(1), 59-90.
11. Mısır, Emre, Emre Bora, and Berna Binnur Akdede, 2018, Relationship between social-cognitive and social-perceptual aspects of theory of mind and neurocognitive deficits, insight level and schizotypal traits in obsessive-compulsive disorder. *Comprehensive Psychiatry*, *83*, 1-6.
12. Weber, Martin, and Colin F. Camerer, 1998, The disposition effect in securities trading: An experimental analysis, *Journal of Economic Behavior and Organization, 33*, 167–184.
13. Todorov, Alexander, Anesu N. Mandisodza, Amir Goren, and Crystal C. Hall, 2005, Inferences of competence from faces predict election outcomes. *Science, 308*(5728), 1623-1626.
14. Willis, Janine, and Alexander Todorov, 2006, First impressions: Making up your mind after a 100-ms exposure to a face. *Psychological Science, 17*(7), 592-598.
15. Pandey, J., & Griffitt, W. (1974). Attraction and helping. *Bulletin of the Psychonomic Society*, 3(2), 123-124.
16. Marchewka, Artur, Łukasz Żurawski, Katarzyna Jednoróg, and Anna Grabowska, 2014, The Nencki Affective Picture System (NAPS): Introduction to a novel, standardized, wide-range, high-quality, realistic picture database. *Behavior Research Methods, 46*(2), 596-610.
17. Riegel, Monika, Łukasz Żurawski, Małgorzata Wierzba, Abnoss Moslehi, Łukasz Klocek, Marko Horvat, Anna Grabowska, Jarosław Michałowski, Katarzyna Jednoróg, and Artur Marchewka, 2016, Characterization of the Nencki Affective Picture System by discrete emotional categories (NAPS BE). *Behavior Research Methods, 48*(2), 600-612.
18. Orehek, Edward, Amanda L. Forest, and Nicole Barbaro, 2018, A people-as-means approach to interpersonal relationships. *Perspectives on Psychological Science, 13*(3), 373-389.

19. Tobii, 2014, Tobii TX300 Eye Tracker User Manual. Tobii Technology. Available at: https://www.tobiipro.com/siteassets/tobii-pro/user-manuals/tobii-pro-tx300-eye-tracker-user-manual.pdf
20. Komogortsev, Oleg V., Denise V. Gobert, Sampath Jayarathna, Do Hyong Koh, and Sandeep M. Gowda, 2010, Standardization of automated analyses of oculomotor fixation and saccadic behaviors. *IEEE Transactions on Biomedical Engineering*, 57(11) 2635-2645.
21. Lacobucci, Dawn, Steven S. Posavac, Frank R. Kardes, Matthew J. Schneider, and Deidre L. Popovich, 2015, Toward a more nuanced understanding of the statistical properties of a median split, *Journal of Consumer Psychology* 25(4), 652-665.
22. Kahneman, Daniel, 1973, *Attention and effort*. Vol. 1063. Englewood Cliffs, NJ: Prentice-Hall.
23. Rehder, Bob, and Aaron B. Hoffman, 2005, Thirty-something categorization results explained: selective attention, eyetracking, and models of category learning. *Journal of Experimental Psychology: Learning, Memory, and Cognition*, *31*(5), 811.
24. Holmqvist, Kenneth, Marcus Nyström, Richard Andersson, Richard Dewhurst, Halszka Jarodzka, and Joost Van de Weijer, 2011, *Eye Tracking: A Comprehensive Guide to Methods and Measures*. Oxford, UK: Oxford University Press.
25. Stampe, Dave M., 1993, Heuristic filtering and reliable calibration methods for video-based pupil-tracking systems. *Behavior Research Methods, Instruments, & Computers*, 25(2), 137-142.
